# A phase transition for finding needles in nonlinear haystacks with LASSO artificial neural networks

**DOI:** 10.1007/s11222-022-10169-0

**Published:** 2022-10-22

**Authors:** Xiaoyu Ma, Sylvain Sardy, Nick Hengartner, Nikolai Bobenko, Yen Ting Lin

**Affiliations:** 1 Shandong University, Jinan, China; 2 Department of Mathematics, University of Geneva, Geneva, Switzerland; 3 Theoretical Biology and Biophysics Group, Los Alamos National Laboratory, Los Alamos, USA; 4 Information Sciences Group, Los Alamos National Laboratory, Los Alamos, USA

**Keywords:** Model selection, Neural networks, Phase transition, Sparsity, Universal threshold

## Abstract

To fit sparse linear associations, a LASSO sparsity inducing penalty with a single hyperparameter provably allows to recover the important features (needles) with high probability in certain regimes even if the sample size is smaller than the dimension of the input vector (haystack). More recently learners known as artificial neural networks (ANN) have shown great successes in many machine learning tasks, in particular fitting nonlinear associations. Small learning rate, stochastic gradient descent algorithm and large training set help to cope with the explosion in the number of parameters present in deep neural networks. Yet few ANN learners have been developed and studied to find needles in nonlinear haystacks. Driven by a single hyperparameter, our ANN learner, like for sparse linear associations, exhibits a phase transition in the probability of retrieving the needles, which we do not observe with other ANN learners. To select our penalty parameter, we generalize the universal threshold of Donoho and Johnstone (Biometrika 81(3):425–455, 1994) which is a better rule than the conservative (too many false detections) and expensive cross-validation. In the spirit of simulated annealing, we propose a warm-start sparsity inducing algorithm to solve the high-dimensional, non-convex and non-differentiable optimization problem. We perform simulated and real data Monte Carlo experiments to quantify the effectiveness of our approach.

## Introduction

Over the past 10 years, Artificial Neural Networks (ANNs) have become the model of choice for machine learning tasks in many modern applications. Although not completely understood today, the beliefs of the reasons for their success are mathematical, statistical and computational.

From the point-of-view of approximation theory, ANNs approximate well smooth functions. For instance a single hidden layer neural net with a diverging number of neurons is dense in the class of compactly supported continuous functions (Cybenko 1989) and the first error rate derived (Barron 1993) motivates shallow learning (few layers) (Ravishankar et al. 2015; Kostadinov et al. 2018). Some results show that deep learning is superior to shallow learning in the sense that less parameters are needed to achieve the same level of accuracy for a smoothness and compositional class of functions, in which case deep learning avoids the curse of dimensionality; see Poggio et al. (2017) for a review. Grohs et al. (2019) prove that deep neural networks provide information-theoretically optimal approximation of a very wide range of functions used in signal processing. Chen and Chen (1995) and related papers extend the results to wider classes of functions. Approximation bound of sparse neural network, that is with bounded network connectivity, has been studied for instance by Bölcskei et al. (2019) who show a link between the degree of connectivity and the complexity of a function class. Adcock et al. (2022) and Adcock and Dexter (2021) show that deep ANNs compare well to benchmark compressed sensing methods with both exponential rates of convergence for analytic functions, using a penalized square-root $\ell _2$-loss.

In machine learning, the success of ANNs is huge and, in part, can be attributed to their expressiveness or capacity (ability to fit a wide variety of functions). The very large number of parameters and the layer structure of ANNs make them impossible to interpret. ANNs are overparametrized with multiple distinct settings of the parameters leading to the same prediction. So traditional measures of model complexity based on the number of parameters do not apply. This makes understanding and interpreting the predictions challenging. Yet in scientific applications, one often seeks to do just that. In keeping with Occam’s razor, among all the models with similar predictive capability, the one with the smallest number of features should be selected. Statistically, models with fewer features not only are easier to interpret but can produce predictors with good statistical properties because such models disregard useless features that contribute only to higher variance.

Operationally, the model selection paradigm often uses a validation set or cross-validation (in which the data is randomly split, models are built on a training set and predictions are evaluated on a validation set). While conceptually elegant, (cross-)validation sets are of limited use if feature selection is of interest (it tends to select many irrelevant features) (Arlot and Celisse 2010), or if fitting a single model is computationally expensive. ANNs and in particular deep ANNs are computationally expensive to fit, so cross-validation is an expensive way of selecting model complexity. Aiming at good predictive performance on a test set, also known as *generalization*, cross-validation is a poor feature selector as it tends to select too many features. In addition, quadratic prediction error from cross-validation exhibits an unexpected behavior with models of increasing complexity: as expected, the training error always decreases with increasing number of input features, but while the quadratic prediction error on the test set is at first U-shaped (initially decreasing thanks to decreasing bias, and then increasing due to an excess of variance), it then unexpectedly decreases a second time. This phenomenon known as *double descent* has been empirically observed (Advani et al. 2020; Geiger et al. 2019). For least squares estimation regularized by an $\ell _2$ ridge penalty (Hoerl and Kennard 1970), double descent has been mathematically described for two-layer ANNs with random first-layer weights by Mei and Montanari (2021) and Hastie et al. (2019). They show that for high signal-to-noise ratio (SNR) and large sample size, high complexity is optimal for the ridgeless limit estimator of the weights, leading to a smooth and more expressive interpolating learner. In other words, interpolation is good and leads to double descent, which after careful thinking should not be a surprise since the interpolating ANN becomes smoother with increasing number of layers, and therefore better predicts between interpolated raining data. Indeed with high SNR, the signal is almost noiseless, so a smooth interpolating function of the training data shall perform well for future prediction. In noisy regimes (that is with low SNR and small sample size), Mei and Montanari (2021) observe that regularization is needed.

In this paper, we present an alternative to the use of a validation set geared towards identifying important features. Specifically, we develop an automatic feature selection method for simultaneous feature extraction and generalization. For ease of exposition, we present our novel method in the context of regression and classification, noting that the ideas can be ported beyond. Our approach exploits ideas from statistical hypothesis testing that directly focus on identifying significant features, and this without explicitly considering minimizing the generalization error. Similar ideas percolate the statistics literature, see for example Johnstone and Silverman (2004), Chen et al. (1999), Tibshirani (1996) with LASSO, Bühlmann and van de Geer (2011) who propose methods for finding *needles in a haystack* in linear models. In this context, the optimized criterion is not the prediction error, but is the ability to retrieve the needles (i.e., relevant features). Useful criteria include the stringent exact support recovery criterion, and softer criteria such as the false discovery rate (FDR) and true positive rate (TPR).

Of course some regularization methods have already been developed to enforce sparsity to the weights of ANNs. For example, *dropout* leaves out a certain number of neurons to prevent overfitting, which incidentally can be used to perform feature selection (Hinton et al. 2012; Srivastava et al. 2014). Sparse neuron architectures can be achieved by other means: Mollaysa et al. (2017) enforce sparsity based on the Jacobian and Li et al. (2016), Lee et al. (2006), Ranzato et al. (2007), Collins and Kohli (2014); Ma et al. (2019) employ $\ell _1$-based LASSO penalty to induce sparsity. Curci et al. (2021) prune their ANNs based on a metric for neuron importance. Evci et al. (2019) discuss the difficulty of training sparse ANNs. spinn (sparse input neural networks) (Feng and Simon 2019) have a sparsity inducing penalty and is governed by two hyperparameters chosen on a validation set; its improved version spinn-dropout (the former originally published in 2017) adds a dropout mechanism governed by an additional hyperparameter (Ye and Sun 2018). So spinn-dropout is a mix between $\ell _1$ and $\ell _0$ (subset selection) sparsity inducing method, similar to the pruning idea (Carreira-Perpinan and Idelbayev 2018; Chao et al. 2020). Sun et al. (2021) propose a Bayesian neural networks (BNN) learner. None of these learners have been studied through the prism of phase transition in the probability of retrieving features.

All of these sparsity inducing methods suffer from two drawbacks: (1) the selection of the penalty parameter(s) is often partly based on a validation set, therefore geared towards good generalization performance, not feature identification, and some hyperparameters are set to arbitrary values; (2) the ability to recover the “right” features has not been quantified through the prism of a phase transition in the probability of support recovery; spinn, spinn-dropout and BNN consider criteria related to FDR and TPR.

This paper is organized as follows. Section 2 presents the theoretical framework and defines our LASSO ANN learner. Section 2.1 defines the statistical model and notation. Section 2.2 reviews the LASSO sparsity paradigm for linear models and extends it to ANNs. Section 2.3 discusses the choice of activation functions. Section 2.4 derives a selection rule for the penalty parameter, a generalization of the universal threshold (Donoho and Johnstone 1994) to non-convex optimization due to the nonlinearity of ANN models. Section 2.5 discusses optimization issues to solve the non-convex high-dimensional and non-differentiable optimization problem. Section 3 evaluates via simulations the ability of our method to exhibit a phase transition in the probability of exact support recovery for the regression task. Section 4 evaluates with a large number of real data sets the ability of our method to perform feature selection and generalization for the classification task. Section 5 summarizes the findings and points to future developments. Proofs and technical details are given in the Appendix.

## LASSO ANN

### Function estimation model and notation

Suppose *n* pairs of ouput-input data $({{\mathcal {Y}}}, {{\mathcal {X}}})=\{(\textbf{y}_i,\textbf{x}_i) \}_{i=1}^n$ are collected to learn about their association. For example, in some medical applications (see Sect. 4.1), $\textbf{x}\in {{\mathbb {R}}}^{p_1}$ is an input vector of $p_1$ gene expressions and $\textbf{y}$ is any of *m* cancer types that is coded as a one-hot output vector of ${\mathbb R}^{m}$; classification aims at assigning the correct type of cancer given an input vector. In regression, *y* is a scalar ($m=1$), for instance riboflavin production rate in a bacteria (see Sect. 4.2).

To model their stochastic nature, data can be modeled as realizations from the pair of random vectors $(\textbf{Y},\textbf{X})$. We assume the real-valued response $\textbf{Y} \in {{\mathbb {R}}}^{m}$ is related to real-valued feature vector $\textbf{X}\in {\mathbb {R}}^{p_1}$ through the conditional expectation

1 $$\begin{aligned} {{\mathbb {E}}}[\textbf{Y}\mid \textbf{X}=\textbf{x}] = \mu (\textbf{x}), \end{aligned}$$

for some unknown function $\mu : {{\mathbb {R}}}^{p_1} \rightarrow \Gamma \subseteq {{\mathbb {R}}}^m$. In regression, $\Gamma ={{\mathbb {R}}}$ and in classification, $\Gamma =\{{\varvec{\pi }}\in ({{\mathbb {R}}}^+)^m: \sum _{k=1}^m \pi _k=1\}$, where $\pi _k$ is the probability of belonging to class *k*.

Many learners have been proposed to model the association $\mu$ between input and output. A recent approach that is attracting considerable attention models $\mu$ as a standard fully connected ANN with *l* layers

2 $$\begin{aligned} \mu _{\varvec{\theta }}(\textbf{x})= S_l \circ \cdots \circ S_1\left( \textbf{x}\right) , \end{aligned}$$

where ${\varvec{\theta }}$ are the parameters (see (5)) indexing the ANN, and letting $\textbf{u}=\textbf{x}$ at the first layer, the nonlinear functions $S_k(\textbf{u})=\sigma (\textbf{b}_k + W_k \textbf{u})$ maps the $p_k\times 1$ vector $\textbf{u}$ into a $p_{k+1}\times 1$ latent vector obtained by applying an activation function $\sigma$ component-wise, for each layer $k \in \{1,\ldots , l-1\}$. The vectors $\textbf{b}_k$ are commonly named “biases.” The matrix of weights $W_k$ is $p_{k+1} \times p_k$ and the operation $+$ is the broadcasting operation.

The last layer $k=l$ has two requirements. First we must have $p_{l+1}=m$ to match the output dimension, so the last function is $S_l(\textbf{u})=G(\textbf{c}+W_l \textbf{u})$ where $W_l$ is $m \times p_l$ and the intercept vector $\textbf{c}\in {{\mathbb {R}}}^{m}$. Second the function $G: {{\mathbb {R}}}^{m}\rightarrow \Gamma$ is a link function that maps ${{\mathbb {R}}}^{m}$ into the parameter space $\Gamma$. Commonly used link functions for classification are

3 $$\begin{aligned} G(\textbf{u})= & {} \left( \frac{\exp \{u_1\}}{\sum _{k=1}^{m}\exp \{u_k\}}, \ldots , \frac{\exp \{u_m\}}{\sum _{k=1}^{m}\exp \{u_k\}}\right) ^{\textrm{T}} \end{aligned}$$

4 $$\begin{aligned} G(\textbf{u})= & {} \left( \frac{\exp \{u_1\}}{\sum _{k=1}^{m-1}\exp \{u_k\} +1}, \ldots , \frac{\exp \{u_{m-1}\}}{\sum _{k=1}^{m-1}\exp \{u_k\}+1},\right. \nonumber \\{} & {} \quad \times \left. \frac{1}{\sum _{k=1}^{m-1}\exp \{u_k\}+1}\right) ^\textrm{T} \end{aligned}$$

respectively called Softmax and multiclass-Logit. For regression, $G(u)=u$.

The parameters indexing the neural network are therefore

5 $$\begin{aligned} {\varvec{\theta }}=(( W_1, \textbf{b}_1, \ldots , \textbf{b}_{l-1}), (W_2, \ldots , W_l,\textbf{c}))=:({\varvec{\theta }}_1, {\varvec{\theta }}_2) \end{aligned}$$

for a total of $\gamma =\sum _{k=1}^l p_{k+1}(p_k+1)$ parameters. The following property is straightforward to prove, but is crucial for our methodology; it is the reason for splitting ${\varvec{\theta }}$ into ${\varvec{\theta }}_1$ and ${\varvec{\theta }}_2$.

#### Property 1

Assuming the activation function satisfies $\sigma (0)=0$, then setting ${\varvec{\theta }}_1=\textbf{0}$ implies $\mu _{\varvec{\theta }}(\textbf{x})$ is the constant function $\mu (\textbf{x})=G(\textbf{c})$ for all $\textbf{x} \in {\mathbb {R}}^{p_1}$.

We believe that only a few features in the $p_1$-long input vector $\textbf{x}$ carry information to predict the output. For many medical data treated in Sect. 4 for instance, the input is a vector of hundreds of gene expressions, and genetic aims to identify the ones having an effect on the output. So our main goal while estimating ${\varvec{\theta }}$ is to find needles in the haystack by selecting a subset of the $p_1$-long inputs by setting some entries of ${\varvec{\theta }}_1$ to zero. Feature selection has been extensively studied for linear associations, showing a phase transition between regimes where features can be retrieved with probability near one to regimes where the probability of retrieving the features is essentially zero. Our goal is to investigate such a phase transition with ANN learners to retrieve features in nonlinear associations.

### Sparse estimation

Finding needles amounts to setting some weights to non-zero values corresponding to features in $\textbf{x}$ that have predictive information. So we seek sparsity in the first layer on the weights $W_1$. For the other layers, large weights in a layer could compensate small weights in the next layer, so we bound them by forcing unit $\ell _2$-norm; instead, Feng and Simon (2019) and Ye and Sun (2018) take the approach of a ridge penalty controlled by an additional hyperparameter fixed to the arbitrary value of 0.0001. Instead we slightly modify the nonlinear terms in (2) and define the $j\text {th}$ nonlinear function $S_{k,j}$ in layer *k* as

6 $$\begin{aligned} S_{k,j}(\textbf{u})= \left\{ \begin{array}{ll} \sigma \left( \textbf{b}_1^{(j)} + \langle \textbf{w}_1^{(j)}, \textbf{u} \rangle \right) &{} k=1\\ \sigma \left( \textbf{b}_k^{(j)} +\frac{ \langle \textbf{w}_k^{(j)}, \textbf{u} \rangle }{\left\| \textbf{w}_k^{(j)} \right\| _2} \right) &{} 1< k <l \\ G\left( \textbf{c}+ \frac{ \langle \textbf{w}_k^{(j)},\textbf{u} \rangle }{\left\| \textbf{w}_k^{(j)}\right\| _2}\right) &{} k=l \end{array} \right. , \quad j \in \{1,\ldots ,p_{k+1}\}, \nonumber \\ \end{aligned}$$

where $\textbf{w}_k^{(j)}$ is the $j\text {th}$ row of $W_k$. At the last layer ($k=l$), $\textbf{c}$ plays the role of an intercept.

Sparsity in the first layer allows interpretability. To enforce sparsity and control overfitting, we take the approach inspired by LASSO of minimizing a compromise between a measure ${{\mathcal {L}}}_n$ of closeness to the data and a measure of sparsity *P*. Owing to Property 1, we estimate the parameters ${\varvec{\theta }}=({\varvec{\theta }}_1, {\varvec{\theta }}_2)$ defined in (5) by aiming the best local minimum

7 $$\begin{aligned} \hat{\varvec{\theta }}_\lambda {\in } \arg \min _{ {\varvec{\theta }}\in {{\mathbb {R}}}^\gamma } {{\mathcal {L}}}_n ({{\mathcal {Y}}} , {\mu }_{\varvec{\theta }}( {{\mathcal {X}}})) + \lambda \ P({\varvec{\theta }}_1) \end{aligned}$$

found by a numerical scheme, where $\lambda >0$ is the regularization parameter of the procedure and *P* is sparsity-inducing penalty (Bach et al. 2011). We stress that our method is driven by a single regularization parameter $\lambda$, as opposed to other methods that use two or three hyperparameters (Ye and Sun 2018; Feng and Simon 2019; Sun et al. 2021).

Common loss functions between training responses ${{\mathcal {Y}}}$ and predicted values ${\mu }_{\varvec{\theta }}( {{\mathcal {X}}})$ include: for *m*-class classification the cross-entropy loss ${{\mathcal {L}}}_n ({{\mathcal {Y}}} , {\mu }_{\varvec{\theta }}( {{\mathcal {X}}}))=\sum _{i=1}^n \textbf{y}_i^{\textrm{T}}\log \mu _{\varvec{\theta }}(\textbf{x}_i)$, where the $\log$ function is applied component-wise to the *m*-long vectors $\textbf{y}_i$ and $\mu _{\varvec{\theta }}(\textbf{x}_i)$; for regression, we use ${{\mathcal {L}}}_n ({{\mathcal {Y}}} , {\mu }_{\varvec{\theta }}( {{\mathcal {X}}}))=(\sum _{i=1}^n ( {y}_i- {\mu }_{\varvec{\theta }}( \textbf{x}_i))^2)^{1/2}$ for reason that will become clear, although its squared version is more often used.

A common sparsity-inducing penalty used by waveshrink (Donoho and Johnstone 1994) and LASSO (Tibshirani 1996) for $q=1$ and group-LASSO (Yuan and Lin 2006) for $q=2$ is

8 $$\begin{aligned} P({\varvec{\theta }}_1)= \sum _{j=1}^{p_1}\Vert \textbf{w}_{1,j}\Vert _q + \sum _{k=1}^{l-1} \Vert \textbf{b}_k \Vert _q , \end{aligned}$$

where $\textbf{w}_{1,j}$ is the $j\text {th}$ column of $W_1$. The choice $q=2$ forces the $j\text {th}$ feature to be either on or off across all neurons. The former is more flexible since a feature can be on in a neuron and off in another, so, in the sequel, we use $q=1$. The reason for penalizing the biases is that the gradient of the loss function with respect to the biases evaluated at zero is zero and that the Hessian is only positive semi-definite (see Appendix C); so a local minimum would not be guaranteed without a penalty on the biases.

ANNs are flexible in the sense that they can fit nonlinear associations. A more rigid and older class of models that has been extensively studied is the class of linear models

9 $$\begin{aligned} \mu _{\varvec{\theta }}^{\textrm{lin}}(\textbf{x})= c+\sum _{j=1}^{p_1} \beta _j x_j, \end{aligned}$$

where here the set of parameters ${\varvec{\theta }}=(\beta _1, \ldots , \beta _{p_1}, c)=:({\varvec{\theta }}_1, c)$ is assumed *s*-sparse, that is only *s* entries in ${\varvec{\theta }}_1$ are different from zero. Here again, like for $W_1$ in ANNs, a non-zero entry in ${\varvec{\theta }}_1$ corresponds to an entry in the input vector $\textbf{x}$ that is relevant to predict the response. For a properly chosen penalty parameter $\lambda$, LASSO has the remarkable property of retrieving the non-zero entries of ${\varvec{\theta }}_1$ in certain regimes (that depend on *n*, $p_1$, SNR, training locations ${{\mathcal {X}}}$ and amount *s* of sparsity), as studied in the noiseless and noisy scenarios by Candès and Tao (2005), Donoho (2006), Donoho et al. (2011), and Bühlmann and van de Geer (2011), for instance. In particular, the value of $\lambda$ must bound the sup-norm of the gradient of the empirical loss at zero with high probability when ${\varvec{\theta }}_1=\textbf{0}$ for LASSO to satisfy oracle inequalities. For linear models in wavelet denoising theory (Donoho and Johnstone 1994), this approach leads to an asymptotic minimax property.

Our contribution is to extend the linear methodology to ANNs, and to investigate how well our extension leads to a phase transition to discover nonlinear lower-dimensional structures in the data.

### Choice of activation functions

Since the weights from level two and higher are bounded on the $\ell _2$-ball of unit radius (6), we require the activation function $\sigma \in {{\mathcal {C}}}^2({{\mathbb {R}}})$ to be unbounded. For reasons related to Property 1 and the choice of the hyperparameter $\lambda$ based on the zero-thresholding function proportional to $\sigma '(0)$ (see (13) and (14)), we require

10 $$\begin{aligned} \sigma (0)=0 \quad \textrm{and} \quad \sigma '(0)>0. \end{aligned}$$

The centered softplus function $\sigma _\textrm{softplus}(u)=\log (1+\exp (u))-\log (2)$ for example satisfies this requirement. The ReLU (Rectified Linear Unit) function $\sigma _{\textrm{ReLU}}(u)=\max (u,0)$ does not because not differentiable at zero.

A legitimate question for a statistician is to ask whether ANNs can retrieve interactions between covariates. Projection pursuit models (Friedman and Stuetzle 1981) have this ability, which additive models do not have. Thanks to ANNs property of being dense in smooth function spaces, the answer is yes, but with a large number of neurons when conventional activation functions like softplus and ReLU are used. With the family of activation functions now defined, a *k*-way interaction can be written with a sparse ANN.

#### Definition 1

The smooth activation rescaled dictionary is the collection of activation functions defined by

11 $$\begin{aligned} \sigma _{M,u_0,k}(u)= & {} \frac{1}{k}(f(u)^k-f(0)^k) \quad \textrm{with} \quad \nonumber \\ f(u)= & {} \frac{1}{M}\log (1+\exp \{M (u+u_0)\}) \end{aligned}$$

indexed by $M>0, u_0>0, k >0$. For $u_0=1$ the dictionary is rescaled in the sense that $\lim _{M\rightarrow \infty }\sigma '_{M,u_0,k}(0)=1$.

Suppose for instance the association is a single two-way interaction, that is $\mu (x)=x_i x_j$ for some pair (*i*, *j*), then with 6 neurons we have $x_i x_j=-1+ \sigma _{\infty ,1,2}(x_i+x_j-1)+ \sigma _{\infty ,1,2}(-x_i-x_j-1)- \sigma _{\infty ,1,2}(x_i-1)- \sigma _{\infty ,1,2}(-x_i-1)- \sigma _{\infty ,1,2}(x_j-1)- \sigma _{\infty ,1,2}(-x_j-1)$ since $x^2/2=1+ \sigma _{\infty ,1,2}(x-1)+ \sigma _{\infty ,1,2}(-x-1)$. When the ANN model employs both linear and quadratic smooth ReLU (shifted by one), selecting neurons with $\sigma _{M,1,k}$ with $k=2$ and *M* large reveals potential interactions.

The proposed activation functions have some basic properties. They satisfy requirements (10). For finite *M*, $\sigma _{M,u_0,j}\in {{\mathcal {C}}}^\infty$. For $(u_0,k)=(0,1)$, the family includes two important activation functions: softplus for $M=1$ and ReLU as *M* tends to infinity. Using ReLU is prohibited with our method; if one likes the shape of ReLU, then one gets a smooth approximation of ReLU choosing a large *M* (say $M=20$). Moreover since $\lim _{M\rightarrow \infty }\sigma '_{M,u_0,k}(0)=u_0^{k-1}$, choosing $u_0=1$ scales activation functions across *k*’s in the sense that their derivatives at zero are asymptotically (as $M\rightarrow \infty$) equal to one for all *k*; this is a desired property in our methodology since the zero-thresholding functions (defined in Theorem 1 and derived in Theorem 3 below) are proportional to $\sigma '(0)$. Moreover, since sparsity is of interest, zero is a region where the cost function ought to be smooth for optimization purposes; hence choosing $u_0=1$ also makes the smoothness of the loss function bounded at zero since $\sigma _{M,1,1}''(0)=M/\exp (M)$ while $\sigma _{M,0,1}''(0)=M/4$ (which reflects that ReLU is not differentiable at zero).

### Selection of penalty $\lambda$

The proposed choice of $\lambda$ is based on Property 1. It shows that fitting a constant function is achieved by choosing $\lambda$ large enough to set the penalized parameters ${\varvec{\theta }}_1$ to zero when solving the penalized cost function (7). For convex loss functions and linear models, the quantile universal threshold (Giacobino et al. 2017) achieves this goal with high probability under the null model that the underlying function is constant. This specific value $\lambda _{\textrm{QUT}}$ has good properties for model selection outside the null model as well (Donoho and Johnstone 1994; Donoho et al. 1995; Bühlmann and van de Geer 2011). The quantile universal threshold has so far been developed and employed for cost functions that are convex in the parameters, hence guaranteeing that any local minimum is also global. The cost function in (7) is not convex for ANN models, so we extend the quantile universal threshold by creating with high probability a local minimum at the sparse point of interest ${\varvec{\theta }}_{1}=\textbf{0}$. This can be achieved thanks to the penalty term $\lambda \ P({\varvec{\theta }}_{1})$ that is part of the cost function in (7), provided $\lambda$ is large enough. The following theorem derives an expression for the zero-thresholding function that gives the smallest $\lambda$ that guarantees a minimum with $\hat{\varvec{\theta }}_1=\textbf{0}$.

#### Theorem 1

For given output–input data $({{\mathcal {Y}}}, {{\mathcal {X}}})$, consider the optimization problem (7) with $P({\varvec{\theta }}_{1})$ defined in (8) with $q=1$, activation function $\sigma \in {{\mathcal {C}}}^2({{\mathbb {R}}})$ and loss function ${{\mathcal {L}}}_n\in {{\mathcal {C}}}^2(\Gamma ^n)$ such that $\hat{\textbf{c}}= \arg \min _{ \textbf{c}\in {{\mathbb {R}}}^m} {{\mathcal {L}}}_n({{\mathcal {Y}}},G(\textbf{c}))$ exists. Let ${\varvec{\theta }}^0=(\textbf{0}_{p_2\times p_1}, W_2 , \ldots , W_l,\hat{\textbf{c}})$ with arbitrary values $W_{k}$ for layers 2 to *l*. Define ${ g}_0({{\mathcal {Y}}}, {{\mathcal {X}}}, {\varvec{\theta }}^0)=\nabla _{{\varvec{\theta }}_{1}} {{\mathcal {L}}}_n(\mathcal{Y},{\varvec{\mu }}_{{\varvec{\theta }}^0}({{\mathcal {X}}}))$. For any $\lambda$ larger than the zero-thresholding function $\lambda _0({{\mathcal {Y}}}, {{\mathcal {X}}})=\sup _{(W_2 \ldots , W_{l})}\Vert { g}_0({{\mathcal {Y}}}, {{\mathcal {X}}}, {\varvec{\theta }}^0) \Vert _\infty$, there is a local minimum to (7) with $(\hat{\varvec{\theta }}_{1,\lambda }, \hat{\textbf{c}}_\lambda )=(\textbf{0}, \hat{\textbf{c}})$.

The proof of Theorem 1 is provided in the appendix; it can be made more general for $q\ge 1$ using Hölder’s inequality. The estimate $\hat{\textbf{c}}$ often has a closed form expression; in regression for instance, if the loss function between ${{\mathcal {Y}}}\in {{\mathbb {R}}}^n$ and $c \textbf{1}$ with $c\in {{\mathbb {R}}}$ and $\textbf{1} \in {{\mathbb {R}}}^n$ is $\mathcal{L}_n({{\mathcal {Y}}},c\textbf{1}) = \Vert {{\mathcal {Y}}}-c \textbf{1}\Vert _2$, then ${{\hat{c}}}=\bar{{\mathcal {Y}}}$, the average of the responses. Based on $\lambda _0({{\mathcal {Y}}}, {{\mathcal {X}}})$, the following theorem extends the universal threshold to non-convex cost functions.

#### Theorem 2

Given training inputs ${{\mathcal {X}}}$, define the random set of outputs ${{\mathcal {Y}}}_0$ generated from (1) with $\mu (\mathcal X)=\mu _{\varvec{\theta }}({{\mathcal {X}}})$ defined in (2) for any activation function satisfying (10) under the null hypothesis $H_0: {\varvec{\theta }}_{1}=\textbf{0}$, that is $H_0: \mu _{\varvec{\theta }}=\textbf{c}$ is a constant function. Letting the random variable $\Lambda =\lambda _0({{\mathcal {Y}}}_0, \mathcal{X})$ and $F_\Lambda$ be the distribution function of $\Lambda$, the quantile universal threshold is $\lambda _\textrm{QUT}=F^{-1}_\Lambda (1-\alpha )$ for a small value of $\alpha$. It satisfies that

12 $$\begin{aligned}{} & {} {{\mathbb {P}}}_{H_0}(\text{ there } \text{ exists } \text{ a } \text{ local } \text{ minimum } \text{ to }\,(7)\hbox { such that } \mu _{\hat{\varvec{\theta }}_{\lambda _{\textrm{QUT}}}}\nonumber \\{} & {} { is constant})\ge 1-\alpha . \end{aligned}$$

A proof of Theorem 2 can be found in Giacobino et al. (2017). The law of $\Lambda$ is unknown but can be easily estimated by Monte Carlo simulation, provided there exists a closed form expression for the zero-thresholding function $\lambda _0({{\mathcal {Y}}}, {{\mathcal {X}}})=\sup _{(W_2 \ldots , W_{l})}\Vert { g}_0({{\mathcal {Y}}}, {{\mathcal {X}}}, {\varvec{\theta }}^0) \Vert _\infty$. The following theorem states a simple expression for $\lambda _0(\mathcal{Y}, {{\mathcal {X}}})$ in two important cases: classification and regression.

#### Theorem 3

Consider a fully connected *l*-layer ANN employing a differentiable activation function $\sigma$ and let $\tau _l=\sqrt{\Pi _{j=3}^l p_{j}}$ for $l\ge 3$, $\tau _2=1$, ${{\mathcal {Y}}}_\bullet ={{\mathcal {Y}}}-\textbf{1}_{n}\bar{{\mathcal {Y}}}$ and $\Vert A\Vert _\infty =\max _{j=1,\ldots ,p} \sum _{i=1}^k |a_{ji}|$ for a $p\times k$ matrix *A*.

- In classification, using the cross-entropy ${{\mathcal {L}}}_n ({{\mathcal {Y}}} , {\mu }_{\varvec{\theta }}( {{\mathcal {X}}}))=\sum _{i=1}^n \textbf{y}_i^{\textrm{T}}\log \mu _\theta (\textbf{x}_i)$ and for the Softmax link function *G* in (3), we have
  13 $$\begin{aligned} \lambda _0({{\mathcal {Y}}}, {{\mathcal {X}}}) = \tau _l \sigma '(0)^{l-1} \Vert \mathcal{X}^{\textrm{T}} {{\mathcal {Y}}}_\bullet \Vert _\infty ; \end{aligned}$$
- In regression, for ${{\mathcal {L}}}_n=\Vert {{\mathcal {Y}}} - \mu _{ \varvec{\theta }}({{\mathcal {X}}}) \Vert _2$, we have
  14 $$\begin{aligned} \lambda _0({{\mathcal {Y}}}, {{\mathcal {X}}}) = \tau _l \sigma '(0)^{l-1} \frac{\Vert {{\mathcal {X}}}^{\textrm{T}} {{\mathcal {Y}}}_\bullet \Vert _\infty }{\Vert \mathcal{Y}_\bullet \Vert _2} . \end{aligned}$$

Theorem 2 states that the choice of $\lambda$ is simply an upper quantile of the random variable $\Lambda =\lambda _0(\mathcal{Y}_0, {{\mathcal {X}}})$, where ${{\mathcal {Y}}}_0$ is the distribution of the response under the null distribution that ${\varvec{\theta }}_1=\textbf{0}$. The upper quantile of $\Lambda$ can be easily estimated by Monte-Carlo simulation.

In regression and assuming Gaussian errors, the null distribution is ${{\mathcal {Y}}}_0 \sim \textrm{N}(c \textbf{1}, \xi ^2 I_n)$. Both the constant *c* and $\xi ^2$ are unknown however, and $\xi ^2$ is difficult to estimate in high dimension. Fortunately, one observes first that (14) involves only the mean centered responses $\mathcal{Y}_\bullet$ and therefore do not dependent on *c*. Second, both numerator and denominator are proportional to $\xi$. Consequently, $\Lambda$ is a pivotal random variable in the Gaussian case. Knowledge of *c* and $\xi ^2$ are therefore not required to derive our choice of hyperparameter $\lambda _{\textrm{QUT}}$. This well-known fact inspired by square-root LASSO (Belloni et al. 2011) motivates the use of ${{\mathcal {L}}}_n=\Vert {{\mathcal {Y}}} - \mu _{ \varvec{\theta }}({{\mathcal {X}}}) \Vert _2$ rather than ${{\mathcal {L}}}_n=\Vert {{\mathcal {Y}}} - \mu _{ \varvec{\theta }}({{\mathcal {X}}}) \Vert _2^2$.

In classification, the null distribution is ${{\mathcal {Y}}}_0 \sim \textrm{Multinomial}(n, {\varvec{\pi }}=G(\textbf{c}))$. The constant vector $\textbf{c}$ is unknown and the random variable $\Lambda$ with $\lambda _0$ defined in (13) is not pivotal. Moreover Holland (1973) proved no covariance stabilizing transformation exists for the trinomial distribution. So the approach we take is to assume the training outputs ${{\mathcal {Y}}}$ reflect the proportion of classes in future samples seeking class prediction. So if $\hat{\varvec{\pi }}$ are the proportions of classes in the training set, then the null distribution is ${{\mathcal {Y}}}_0 \sim \textrm{Multinomial}(n, {\varvec{\pi }}={\hat{\varvec{\pi }}})$. The quantile universal threshold derived under this null hypothesis is appropriate if future data come from the same distribution, which is a reasonable assumption.

### Computational cost for LASSO ANN

For a given $\lambda$, we solve (7) first by steepest descent with a small learning rate, and then employ a proximal method to refine the minimum by exactly setting to zero some entries of ${{\hat{W}}}_{1,\lambda _{\textrm{QUT}}}$ (Beck and Teboulle 2009; Bach et al. 2011).

Solving (7) directly for the prescribed $\lambda =\lambda _{\textrm{QUT}}$ risks getting trapped at some poor local minimum. Instead, inspired by simulated annealing and warm start, we avoid thresholding too hardly at first and possibly missing important features by solving (7) for an increasing sequence of $\lambda$’s tending to $\lambda =\lambda _{\textrm{QUT}}$, namely $\lambda _{k+1}=\exp (k)/(1+\exp (k))\lambda _{\textrm{QUT}}$ for $k\in \{-1,0,\ldots ,4\}$. Taking as initial parameter values the solution corresponding to the previous $\lambda _k$ leads to a sequence of sparser approximating solutions until solving for $\lambda _{\textrm{QUT}}$ at the last step. We do not perform multiple starts.

The computational cost is low. It requires solving (7) approximately on the small grid of $\lambda$’s tending to $\lambda _{\textrm{QUT}}$ using the warm start to finally solve (7) precisely for $\lambda _{\textrm{QUT}}$. Calculating $\lambda _{\textrm{QUT}}$ is also cost efficient (and highly parallelizable) since it is based on an *M*-sample Monte Carlo that calculates *M* gradients $\{{ g}_0({ y}_k, { X}, {\varvec{\theta }}_0)\}_{k=1}^M$ using backpropagation (Rumelhart et al. 1986) for *M* Gaussian samples $\{y_k\}_{k=1}^M$ under $H_0$; see Theorems 1 and 2 for details. Using *V*-fold cross-validation instead would require solving  (7) a total of $V*L$ times, where *L* is the number of $\lambda$’s visited until finding a (hopefully global) minimum to the cross-validation function. Using a validation set reduces complexity by a factor *V*, at the cost of using data to validate. Instead, our quantile universal threshold approach does not require a validation set.

The phase transition property achieved with LASSO ANN shows its stability to repeatedly identify the same relevant features over many training sets. Yet, Bastounis et al. (2021a, 2021b) and Colbrook et al. (2022) have proved that stability and generability do not exist together for ANN as well as LASSO ANN learners, showing some limitation of the method.

## Regression simulation study

The regression problem is model (1) for scalar output ($m=1$), Gaussian additive noise and (unknown) standard deviation, here chosen $\xi =1$. To evaluate the ability to retrieve needles in a haystack, the true associations $\mu$ is written as sparse ANNs that uses only *s* of the $p_1$ entries in the inputs $\textbf{x}$. We say an association $\mu$ is *s*-sparse when it uses only *s* input entries, that is $s=|S|$ where $S=\{j\mid x_j \text{ carries } \text{ information } \}$ in the association $\mu$. A sparse ANN learner estimates which inputs are relevant by estimating the support with

15 $$\begin{aligned} {{\hat{S}}}=\{j \mid \Vert \hat{\textbf{w}}_{1,j}\Vert > \epsilon \}, \end{aligned}$$

where $\hat{\textbf{w}}_{1,j}$ is the $j\text {th}$ column of the estimated weights ${{\hat{W}}}_1$ at the first layer. Likewise for linear model (9), the support is estimated with ${{\hat{S}}}=\{j \mid {\hat{\beta }}_j \ne 0\}$.

Since we employ a precise thresholding algorithm to solve (7), we use $\epsilon =0$ to determine ${{\hat{S}}}$ in (15); other methods apply a hard thresholding step with a second hyperparameter $\epsilon$ to get rid of small values. Our method could be improved by using $\epsilon$ as another hyperparameter, but our aim is to investigate a phase transition with LASSO ANN, so we consider a single hyperparameter $\lambda$, and show that choosing $\lambda =\lambda _{\textrm{QUT}}$ leads to a phase transition.

To quantify the performance of the tested methods, we use four criteria: the probability of exact support recovery $\textrm{PESR}={{\mathbb {P}}}({{\hat{S}}}=S)$, the true positive rate $\textrm{TPR}={{\mathbb {E}}}\left( \frac{|S \bigcap {{\hat{S}}}|}{|S|} \right)$, the false discovery rate $\textrm{FDR}={{\mathbb {E}}}\left( \frac{| \bar{S} \bigcap {{\hat{S}}}|}{|{{\hat{S}}}| \vee 1} \right)$, and the generalization or predictive error $\textrm{PE}^2={\mathbb E}(\mu (X)-{\hat{\mu }}(X))^2$. Although stringent, the PESR criterion reaches values near one in certain regimes. In fact, a phase transition has been observed for linear models: PESR is near one when the complexity parameter *s* is small, and PESR suddenly decreases to zero when *s* becomes larger (Candès and Tao 2005; Donoho 2006). One wonders whether this phenomenon is also present for nonlinear models, which we are investigating below. A high TPR with low FDR is also of interest, but is a criterion less strict than having high PESR.

We consider five learners: a standard ANN with keras available in TensorFlow (with its optimizer=‘sgd’ option) with no sparsity inducing mechanism; spinn (sparse input neural networks) (Feng and Simon 2019) with sparsity mechanisms governed by two hyperparameters chosen on a validation set; spinn-dropout (which Python code was kindly provided to us by the first author) (Ye and Sun 2018) with sparsity inducing mechanisms (including dropout) governed by three hyperparameters chosen on a validation set; Bayesian neural networks (BNN) (Sun et al. 2021) driven by three hyperparameters; and our LASSO ANN with a sparsity inducing penalty governed by a single hyperparameter chosen by the same QUT principle (and no validation set required).

For LASSO ANN we use two to four-layer ANNs with the arbitrary choices of $(p_2,p_3,p_4)=(20, 10, 5)$ (small values because the sample size is small, and decreasing sequence because many practitioners choose such an architecture), activation function $\sigma _{20,1,1}$ defined in (11) ($M=20$ to have an approximation error of ReLU negligible compared to the noise level $\xi$) and the $\ell _1$-LASSO penalty. spinn, spinn-dropout and BNN use ReLU.

The ReLU activation function allows to sparsely write a linear association (Sect.  3.1) and the nonlinear absolute value function (Sect. 3.2). With Monte-Carlo simulations to estimate PESR, TPR, FDR and PE in two different settings, we investigate the behavior of these four criteria as a function of the model complexity parameter *s*, for fixed sample size *n* and signal to noise ratio governed by $(\xi , \theta )$. The first simulation assumes a sparse linear association and compares LASSO ANN to the benchmark square-root LASSO for linear models. The second simulation assumes a sparse nonlinear association. These allegedly simple sparse associations reveal a phase transition in the ability of LASSO ANN to retrieve needles in haystacks in a more coherent way than with the more complex (i.e., more than one hyperparameter) spinn, spinn-dropout and BNN learners.

### Linear associations

The linear model (9) is the most commonly used and studied model, so we investigate in this section how LASSO ANN compares to a state-of-the-art method for linear models, here square-root LASSO (Belloni et al. 2011) (using the slim function in the flare library in R). This allows to investigate the impact of the loss of convexity for ANNs.

Assuming the linear association is *s*-sparse, this section compares the ability to retrieve the *s* relevant input entries assuming either a linear model (the benchmark) or a non-linear model using fully connected ANNs. The aim of the Monte Carlo simulation is to investigate:

1. a phase transition with LASSO ANN and if so, how close it is to the phase transition of square-root LASSO which, assuming a linear model, should be difficult to improve upon. We consider two selection rules for $\lambda$ for square-root LASSO: QUT and using a validation set to minimize the predictive error.
2. how the quantile universal threshold $\lambda _{\textrm{QUT}}$ based on (14) performs for LASSO ANN with two, three and four layers.
3. a phase transition with spinn and spinn-dropout. In an attempt to make them comparable to LASSO, we set their parameter controlling the trade-off between LASSO and group-LASSO to a small value so that their penalty is essentially LASSO’s. Like LASSO, spinn and spinn-dropout use a validation set to tune their hyperparameters. Results with their default values are not as good and not reported here.

This experimental setting allows various interesting comparisons: linear versus nonlinear models to retrieve a linear model, and model selection- (QUT) versus validation set-based choice of the hyperparameter(s).

We estimate the PESR criterion of the three methods with a Monte-Carlo simulation with 100 repetitions. Each sample is generated from an *s*-sparse linear model with $s\in \{0,1,2,\ldots ,16\}$, the sample size is $n=100$ from and the dimension of input variables is $p_1=2n$. Donoho and Tanner (2010) studied in the noiseless case the performance of $\ell _1$-regularization as a function of $\delta =n/p_1$ and $\rho =s/n$ (for us, $\delta =1/2$ and $\rho =s/100$) and found a PESR phase transition. To be close to their setting, we assume the input variables are i.i.d. standard Gaussian with a moderate signal-to-noise ratio: the *s* non-zero linear coefficients $\beta _j$ in (9) are all equal to 3 and the standard deviation of the Gaussian noise is $\xi =1$. ANN models with ReLU fits linear models sparsely. Indeed a two-layer ANN with a single activated neuron with *s* non-zero entries in the weights $W_1$ matches the linear function in the convex hull of the data, as stated in the following property.

#### Property 2

Using the ReLU activation function, an *s*-sparse linear function restricted to the convex hull of the *n* data vectors $\{\textbf{x}_i\}_{i=1,\ldots ,n}$ can be written as a two-layer neural network with a single neuron with a row matrix $W_1$ with *s* non-zero entries.

The proof of Property 2 is provided in the appendix. The convex hull includes the *n* observed covariates which enter the square-root $\ell _2$-loss in (7). So the sparsest two-layer ANN model that solves the optimization and that is a linear model in the convex hull of the data has a single neuron. But the ANN fit is no longer linear outside the convex hull, which makes prediction error PE poor outside the convex hull range of the data; we therefore do not report PE for the linear model since the ANN model will have poor performance for test data outside the convex hull of the training data.

**Fig. 1:**
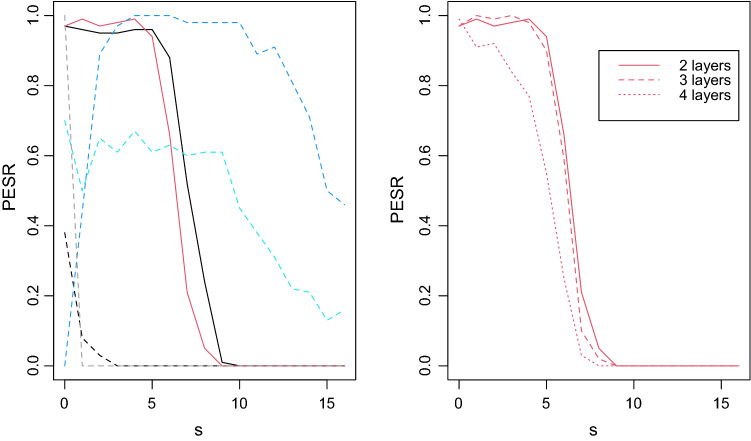
Monte-Carlo simulation results for linear association plotting the estimated probability of exact support recovery (PESR). Left plot: the two black curves assume a linear model while the color curves assume an ANN model; the two blue lines (light for spinn and dark for spinn-dropout) and the grey line (BNN) are governed by more than one hyperparameter while the red line (LASSO ANN) is governed by a single hyperparameter; the two continuous lines (black for square-root LASSO linear and red for LASSO ANN) select the hyperparameter with QUT while the dashed lines require a validation set. Right plot: LASSO ANN with 2 to 4 layers with its hyperparameter based on QUT

Figure 1 summarizes the results of the Monte-Carlo simulation. As in Donoho and Tanner (2010), we observe a PESR phase transition. Surprisingly, little is lost with LASSO ANN (red curve) compared to linear model (black line), showing the good performance of our choice of $\lambda _{\textrm{QUT}}$ and optimization scheme. The linear model based on a validation set (black dashed line) shows poor performance in terms of PESR, as expected. In summary, LASSO ANN compares surprisingly well to the benchmark linear square-root LASSO with QUT by not losing much in terms of PESR. The other three ANNs learners spinn, spinn-dropout and BNN cannot directly be compared to the other since governed by more than one hyperparameter, but, while we observe good PESR for *s* large, their global behavior does not follow the conventional phase transition (that is, no high plateau near one for small *s* and rapidly dropping down to zero with larger *s*); the nonlinear simulation in the next section confirms their non-conventional behaviors. Going back to LASSO ANN, we observe on the right plot of Fig. 1 that using more layers slightly lowers the performance, as expected, but that the choice of $\lambda _{\textrm{QUT}}$ for more layers still leads to a phase transition.

**Fig. 2:**
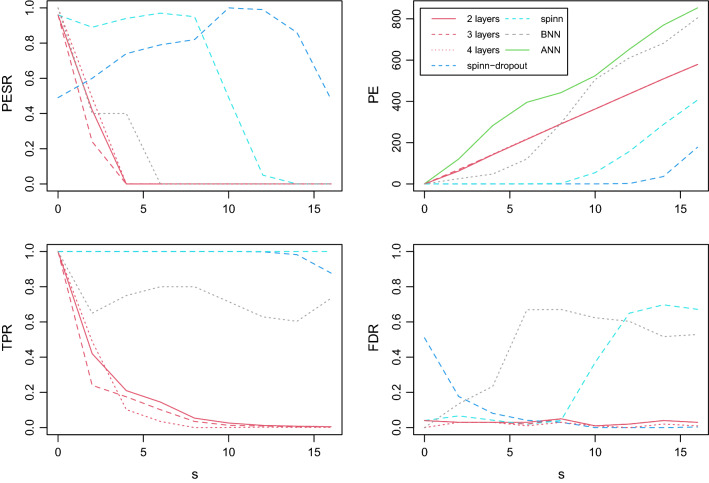
Monte-Carlo simulation results for nonlinear association plotting the estimated probability of exact support recovery (PESR-top left), generalization (PE-top right), true positive rate (TPR-bottom left) and false discovery rate (FDR-bottom right). The red curves are for LASSO ANN with its hyperparameter based on QUT with two (continuous) to four layers (dashed). The two blue lines (light for spinn and dark for spinn-dropout) and and the grey line (BNN) are governed by more than one hyperparameter selected based on a validation set. The green curve is a standard ANN (without sparsity constraint)

### Nonlinear associations

To investigate a phase transition as a function of *s*, we consider *s*-sparse functions of the form $\mu _{\varvec{\theta }}(\textbf{x})= \sum _{i=1}^h 10\cdot |x_{2i}-x_{2i-1}|$ for $h \in \{0,1,\ldots ,8 \}$, which corresponds to *s* needles in a nonlinear haystack with $s\in \{ 0, 2, \ldots , 16 \}$. Because this association is harder to retrieve than the linear one (due to the non-monotone nature of the absolute value function), the haystack is of size $p_1=50$ and the training set is of size $n=500$. This ratio $\delta =n/p_1=10$ seems to be the limit where needles can be recovered with LASSO ANN. The association $\mu _{\varvec{\theta }}(\textbf{x})$ is well approximated by a sparse two-layer ANN employing the smooth activation function $\sigma _{20,1,1}$ and with $c=10s$, $\textbf{w}_{2}=(10\cdot \textbf{1}_{h}^{\textrm{T}}$, $\textbf{0}_{p_2/2-h}^{\textrm{T}},10\cdot \textbf{1}_{h}^\textrm{T}$, $\textbf{0}_{p_2/2-h}^{\textrm{T}})$, $\textbf{b}_{1}=-\textbf{1}_{p_2}$ and16The columns of $W_1$ being sparse, a LASSO is more appropriate than a group-LASSO penalty.

**Table 1:** Some data characteristics (results for data with $\dagger$ are plotted in Fig. 3)

Dataset	Domain	n	${p}_1$	n/${p}_1$	m	Source
Climate	Climate model	540	18	30.0000	2	UCI-MLR
Breast$\dagger$	Breast cancer	569	30	18.9667	2	python sklearn
Wine$\dagger$	Wine	178	13	13.6923	3	python sklearn
Connectionist	Connectionism	208	60	3.4667	2	UCI-MLR
Bearing	Engine noise	952	1024	0.9297	4	CWRU data center
Sorlie	Breast cancer	85	456	0.1864	5	R: datamicroarray
BCI$\_2240^\dagger$	Brain signal	378	2240	0.1688	2	BCI competition
Christensen	Medical	217	1413	0.1536	3	R: datamicroarray
Genes	Cancer RNA	801	12356	0.0648	5	UCI-MLR
Gravier	Breast cancer	168	2905	0.0578	2	R: datamicroarray
Alon	Colon cancer	62	2000	0.0310	2	R: datamicroarray
Khan	Blue cell tumors	63	2308	0.0273	4	R: datamicroarray
Yeoh$\dagger$	Leukemia	248	12625	0.0196	6	R: datamicroarray
Su	Medical	102	5565	0.0183	4	R: datamicroarray
Gordon	Lung cancer	181	12533	0.0144	2	R: datamicroarray
Tian	Myeloma	173	12625	0.0137	2	R: datamicroarray
Shipp	Lymphoma	77	7129	0.0108	2	R: datamicroarray
Golub	Leukemia	72	7129	0.0101	2	R: datamicroarray
Pomeroy	Nervous system	60	7128	0.0084	2	R: datamicroarray
Singh	Prostate cancer	102	12600	0.0081	2	R: datamicroarray
West	Breast cancer	49	7129	0.0069	2	R: datamicroarray
Burczynski	Crohn’s disease	127	22283	0.0057	3	R: datamicroarray
Chin	Breast cancer	118	22215	0.0053	2	R: datamicroarray
Subramanian	Medical	50	10100	0.0050	2	R: datamicroarray
Chowdary	Breast cancer	104	22283	0.0047	2	R: datamicroarray
Borovecki	Medical	31	22283	0.0014	2	R: datamicroarray

**Fig. 3:**
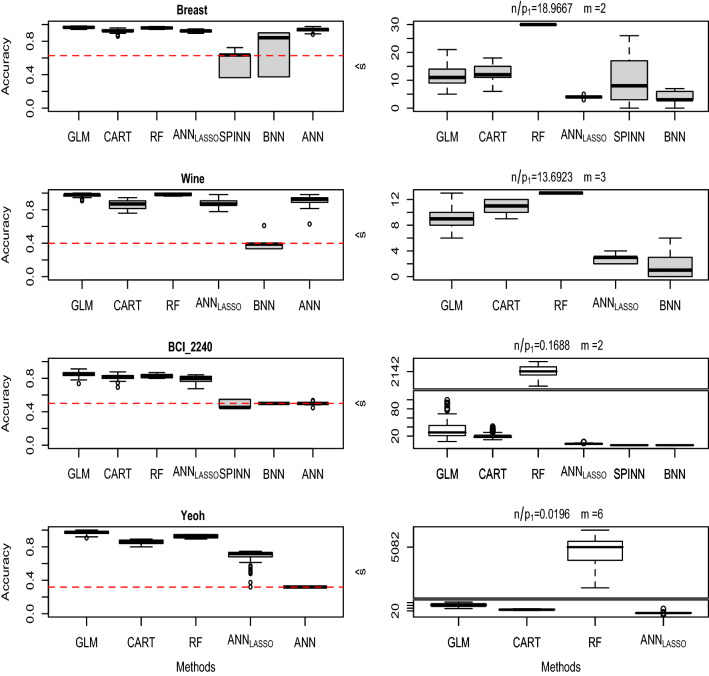
Monte-Carlo simulation results based on four representative data sets, namely, Breast, Wine, BCI$\_$2240, Yeoh of Table 1. The left boxplots are the accuracy results and the right boxplots are the number of selected needles. The horizontal red line is the accuracy by always predicting the most frequent class (that is, without looking at the inputs)

Figure 2 reports the estimated PESR, TPR, FDR and PE criteria as a function of the sparsity level *s*.

We observe that, as for linear models, LASSO ANN (red lines for two to four layers) has a PESR phase transition thanks to a good trade-off between high TPR and low FDR. Moreover LASSO ANN generalizes better in this setting than the off-the-shelf ANN learner (green lines). The other three ANNs learners spinn , spinn-dropout and BNN (light and dark blue, and grey respectively) perform poorly is terms of FDR, but somewhat better in terms of PESR thanks to more than one hyperparameter. The good FDR control of LASSO ANN is striking, in particular at $s=0$ where its value is near $\alpha =0.05$, as mathematically expected, proving the effectiveness of not only QUT but also of the optimization algorithm. Finally, as far as generalization is concerned, the sparsity inducing learners perform better than the conventional ANN learner since the underlying ANN model is indeed sparse. Because LASSO ANN not only selects a sparse model but also shrinks, its predictive performance is not as good as with spinn and spinn-dropout which regularization parameters are selected to generalize well, but LASSO ANN is better than BNN.

### Conclusions of the Monte Carlo simulations

With a single hyperparameter, LASSO ANN has a phase transition for both linear and nonlinear associations and a good FDR control, proving the effectiveness of our quantile universal threshold and optimization scheme. The linear simulation reveals that the impact of the loss of convexity is mild with LASSO ANN since we essentially get the same phase transition as with a linear model. The other ANN learners do not have a conventional phase transition and do not control FDR well; yet, with the help of more hyperparameters selected based on a validation set, they are able to sometimes generalize well.

## Application to real data

### Classification data

The characteristics of 26 classification data sets are listed in Table 1, in particular the sample size *n*, the number of inputs $p_1$ and the number of classes *m*. Most inputs are gene expressions, but there are also FFT preprocessed time series and other types of inputs.

**Fig. 4:**
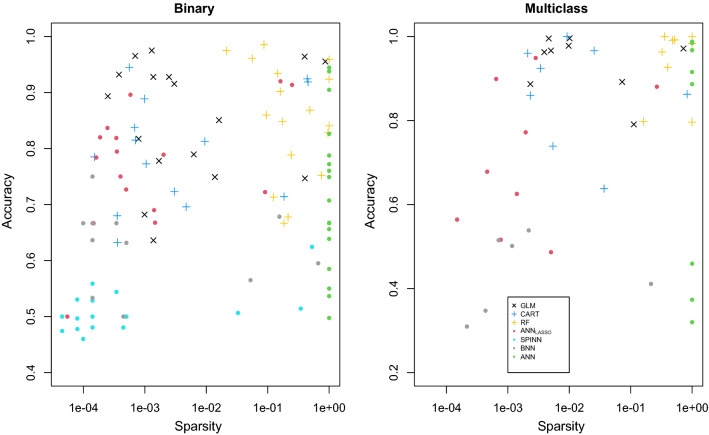
Summary of Monte-Carlo results for all data sets of Table 1. The *x*-axis measures sparsity on a log-scale with $({{\hat{s}}}+1)/(p_1+1)$ and the *y*-axis is accuracy. Ideal points are near the top-left corner of the figure

We randomly split the data into training (70%) and test (30%) sets, repeating the operation 100 times. Figure 3 reports the results for four data sets chosen for their ratios $n/p_1$ and their number of classes *m* (marked with a $\dagger$ in Table 1). The left boxplots of Fig. 3 report classification accuracy, and the right boxplots report the number ${{\hat{s}}}$ of selected needles. High accuracy along with low ${{\hat{s}}}$ reflects good needles selection. The results of all 26 sets are summarized in the scatter plot of Fig. 4.

We train and test the following learners: LASSO GLM with $\lambda$ chosen to minimize 10-fold cross validation (Friedman et al. 2010) in R with glmnet, CART (Breiman et al. 1984) in R with rpart, random forest (Breiman 2001) in R with randomForest, spinn in Python for binary classification (no code for multiclass and for spinn-dropout available), Bayesian neural networks (BNN), standard ANN learner in Python with keras and its optimizer=‘adam’ option, and our LASSO ANN with two layers in Python. Random forest is an ensemble learner that combines CARTs; so the comparison between CART and random forest quantifies the ensembling effect, and the comparison between CART and LASSO ANN is more fair since both are no ensemble learners.

Figure 4 visualizes the accuracy-sparsity trade-off by plotting accuracy versus $({{\hat{s}}}+1)/(p_1+1)$ on a log-scale, so that both axes are on [0, 1]. Learners with points near (0, 1) offer the best trade-off. We were able to apply CART and BNN to only 22 and 17 data sets, respectively, because of memory issues when $p_1$ is too large. Among all ANN-based learners (represented with “o”), LASSO ANN is clearly the best.

The main lesson of this experiment on real data sets is that LASSO ANN offers a good compromise between high accuracy and low number of selected needles. Yet, linear learners are difficult to beat when $n/p_1\ll 1$, which corroborates our findings in regression that the sample size must be large to identify nonlinear associations.

### Regression data

Bühlmann et al. (2014) reported genetic data measuring the expression levels of $p_1= 4088$ genes on $n= 71$ Bacillus subtilis bacteria. The logarithms of gene expression measurements are known to have some strongly correlated genes, which also makes selection difficult. The output is the riboflavin production rate of the bacteria. This is a high-dimensional setting in the sense that the training set is small compared to the size of the haystack. Generalization is not the goal here, but finding the informative genes; the scientific questions are: what genes affect the riboflavin production rate? Is the association linear?

The ground truth is not known here, but LASSO-zero, a conservative method with low false discovery rate (Descloux and Sardy 2021), selects genes 4003 and 2564. Standard LASSO (using cv.glmnet in R) selects 30 genes including 4003 and 2564. Using $p_2=20$ neurons, LASSO ANN finds a single active neuron containing 9 non-zero parameters including genes 4003 and 2564. Feng and Simon (2019) reports 45 important genes with spinn, and running spinn-dropout 100 times (randomly splitting into $70\%$ training and $30\%$ validating) we find an average of 6 genes (in which 4003 and 2564 are rarely present). BNN selects zero genes, in particular because the sample size here is really small. So the answers to the scientific questions are that few genes seem responsible for riboflavin production and that a linear model seems sufficient.

## Conclusion

For finding needles in a nonlinear haystack, LASSO ANN, with a simple principle to select a single hyperparameter, achieves: (1) a phase transition in the probability of exact support recovery and controls well the false discovery rate; (2) a consistent good trade-off between generalization and low number of selected needles whether in regression, binary or multiclass classification with various $n/p_1$ ratios. This makes it a good candidate to discover important features without adding many spurious ones. Our empirical findings call for more theory to mathematically predict the regimes indexed by $(n,\textbf{p},s,\xi ,\theta ,\sigma )$ where feature recovery is highly probable. We also introduce a class of rescaled activation functions $\sigma _{M,u_0,k}$ that can be employed in different neurons.

ANN models are widely used state-of-the-art black boxes. There is a keen interest, especially in scientific applications, to understand the why of model predictions. Sparse encoding automatic feature selection provides a path towards such an understanding.

Our work makes sparse encoding with LASSO ANN closer to practical applications. Its coherent PESR behavior and FDR control make it reliable for finding needles in nonlinear haystacks, but could also be used for other ANN tasks requiring sparsity, e.g., sparse auto-encoding or convolutional ANN (He et al. 2020).

## Reproducible research

Our codes are available at https://github.com/StatisticsL/ANN-LASSO, for BNN at https://github.com/sylydya/Consistent-Sparse-Deep-Learning-Theory-and-Computation, for SPINN binary classification at https://github.com/jjfeng/spinn.

## A Proof of Theorem 1

Let ${W}_1$ be any matrix with $\Vert {W}_1\Vert _1 = 1$ and $\textbf{c}_1\in {\mathbb {R}}^r$ be any vector with $\Vert \textbf{c}_1\Vert _1 = 1$. Let ${{\varvec{\theta }}^\epsilon } =(\epsilon W_1, W_2, \ldots , W_{l},\hat{\textbf{c}}+\epsilon \textbf{c}_1)$ for any $\tilde{\varvec{\theta }}=(W_2, \ldots , W_{l})$.

Since the loss function *l* is twice differentiable with respect to $W_1$ around ${\varvec{\theta }}^0$, applying Taylor’s theorem we have

$$\begin{aligned}{} & {} |{{\mathcal {L}}}_n({{\mathcal {Y}}}, \mu _{{\varvec{\theta }}^\epsilon }({{\mathcal {X}}})) - {{\mathcal {L}}}_n({{\mathcal {Y}}}, \mu _{{\varvec{\theta }}^0}({{\mathcal {X}}}))| \\{} & {} \quad = |\nabla _{W_1} {{\mathcal {L}}}_n ({{\mathcal {Y}}}, \mu _{{\varvec{\theta }}^0}(x)) (\epsilon {W}_1)+\nabla _{\textbf{c}_1} {{\mathcal {L}}}_n({{\mathcal {Y}}}, \mu _{{\varvec{\theta }}^0}({{\mathcal {X}}})) (\epsilon \textbf{c}_1)\\{} & {} \qquad + o(\epsilon ^2 \Vert {W}_1\Vert _1)+ o(\epsilon ^2 \Vert \textbf{c}_1\Vert _1)| \\{} & {} \quad = |\epsilon g_0({{\mathcal {Y}}},{{\mathcal {X}}}, {\varvec{\theta }}^0) {W}_1 +0+ o(\epsilon ^2) |\\{} & {} \qquad \le |\epsilon | \Vert g_0({{\mathcal {Y}}},{{\mathcal {X}}},{\varvec{\theta }}^0)\Vert _\infty + o(\epsilon ^2). \end{aligned}$$

Therefore we get ${{\mathcal {L}}}_n({{\mathcal {Y}}}, \mu _{{\varvec{\theta }}^\epsilon }({{\mathcal {X}}})) \ge {{\mathcal {L}}}_n({{\mathcal {Y}}}, \mu _{{\varvec{\theta }}^0}({{\mathcal {X}}})) - |\epsilon | \Vert g_0(\mathcal{Y},{{\mathcal {X}}},{\varvec{\theta }}^0)\Vert _\infty + o(\epsilon ^2).$

If we assume that $(\lambda - \sup _{\tilde{\varvec{\theta }}}\Vert g_0({{\mathcal {Y}}},{{\mathcal {X}}},{\varvec{\theta }}^0)\Vert _\infty )> C > 0$ (since we normalize layers 2 to *l*, the supremum is finite), it follows that the regularized loss satisfies

$$\begin{aligned} {{\mathcal {L}}}_n({{\mathcal {Y}}}, \mu _{{\varvec{\theta }}^\epsilon }({{\mathcal {X}}}))&+ \lambda \Vert \epsilon {W}_1\Vert _1 \\ {}&\ge {{\mathcal {L}}}_n({{\mathcal {Y}}}, \mu _{{\varvec{\theta }}^0}({{\mathcal {X}}})) \\&\quad + |\epsilon | (\lambda - \Vert g_0({{\mathcal {Y}}},{{\mathcal {X}}},{\varvec{\theta }}^0)\Vert _\infty ) + o(\epsilon ^2) \\&> {{\mathcal {L}}}_n({{\mathcal {Y}}}, \mu _{{\varvec{\theta }}^0}({{\mathcal {X}}})) + |\epsilon | C + o(\epsilon ^2) \\&> {{\mathcal {L}}}_n({{\mathcal {Y}}}, \mu _{{\varvec{\theta }}^0}({{\mathcal {X}}})) \quad \text {for }|\epsilon |\text { small enough.} \end{aligned}$$

Thus the cost function (7) with our choice of $\lambda$ indeed has a local minimum at ${\varvec{\theta }}^0$.

## B Proof of Theorem 3

Let $W_{k;:i}$ be the *i*-th column of $W_{k}$. We consider two tasks:

- Regression with the square-root $\ell _2$-loss
  $$\begin{aligned} \begin{aligned} {{\mathcal {L}}}_n({{\mathcal {Y}}}, \mu _{\varvec{\theta }}({{\mathcal {X}}}))&=\Vert {{\mathcal {Y}}} - \mu _{ \varvec{\theta }}({{\mathcal {X}}}) \Vert _2 =\sqrt{ \sum _{k=1}^{n}\left( y_k-(c+\textbf{w}_{l}\sigma (\textbf{b}_{l-1}+W_{l-1}\sigma (\cdots \sigma (\textbf{b}_{1}+W_1{\varvec{x}}_k))) \right) ^{2}}, \end{aligned} \end{aligned}$$
  where $y_k \in {\mathbb {R}}$, $c \in {\mathbb {R}}$, $\textbf{w}_l \in 1 \times {\mathbb {R}}^{p_l}$, $W_{k} \in {\mathbb {R}}^{p_{k+1} \times p_{k}},k=1,\ldots ,l-1$ and $\textbf{b}_{k}\in {\mathbb {R}}^{p_{k+1} \times 1},k=1,\ldots ,l-1$. At ${\varvec{\theta }_1}=\textbf{0}$, the least squares problem is solved for ${\hat{c}} = \bar{{{\mathcal {Y}}}}$, the average of the training set responses. So we want to evaluate the gradient with respect to ${\varvec{\theta }_1}$ at $({\varvec{\theta }_1}, c)=(\textbf{0}, \bar{{\mathcal {Y}}})$, called condition $L_0$. Some elementary calculation yields
  $$\begin{aligned} \frac{\partial {{\mathcal {L}}}_n({{\mathcal {Y}}}, \mu _{\varvec{\theta }}(\mathcal{X}))}{\partial W_{1;i,j}}\bigg |_{L_0}= & {} \frac{\sigma ^{'}(0)^{l-1}}{\Vert {{\mathcal {Y}}}_\bullet \Vert _2} \left( -\textbf{w}_lW_{l-1}\ldots W_{2;:i}\right) \\{} & {} \sum _{k=1}^{n}(y_k-\bar{\mathcal{Y}})x_{k,j}, \\ \frac{\partial {{\mathcal {L}}}_n({{\mathcal {Y}}}, \mu _{\varvec{\theta }}(\mathcal{X}))}{\partial b_{h;i}}\bigg |_{L_0}= & {} \frac{\sigma ^{'}(0)^{l-h}}{\Vert {{\mathcal {Y}}}_\bullet \Vert _2} \left( -\textbf{w}_l\ldots W_{h+1;:i}\right) \\{} & {} \sum _{k=1}^{n}(y_k-\bar{{{\mathcal {Y}}}}), 1\le h \le (l-2), \\ \frac{\partial {{\mathcal {L}}}_n({{\mathcal {Y}}}, \mu _{\varvec{\theta }}(\mathcal{X}))}{\partial b_{l-1;i}}\bigg |_{L_0}= & {} \frac{\sigma ^{'}(0)}{\Vert {{\mathcal {Y}}}_\bullet \Vert _2} \left( -\textbf{w}_{l;i}\right) \sum _{k=1}^{n}(y_k-\bar{{{\mathcal {Y}}}}), \end{aligned}$$
  leading to
  $$\begin{aligned}{} & {} \nabla _{\varvec{\theta }_1}{{{\mathcal {L}}}_n({{\mathcal {Y}}}, \mu _{\varvec{\theta }^{0}}({{\mathcal {X}}}))} \\{} & {} \quad =\frac{1}{\Vert \mathcal{Y}_\bullet \Vert _2} \begin{pmatrix} \left[ \sigma ^{'}(0)^{l-1}\left( -\textbf{w}_lW_{l-1}\ldots W_{2;:1}\right) \sum _{k=1}^{n}(y_k-\bar{{{\mathcal {Y}}}})x_{k,j}\right] _{j \in \{1,\ldots ,p_1\}}\\ \vdots \\ \left[ \sigma ^{'}(0)^{l-1} \left( -\textbf{w}_lW_{l-1}\ldots W_{2;:p_2}\right) \sum _{k=1}^{n}(y_k-\bar{{{\mathcal {Y}}}})x_{k,j}\right] _{j \in \{1,\ldots ,p_1\}}\\ \left[ \sigma ^{'}(0)^{l-1} \left( -\textbf{w}_l\ldots W_{2;:i}\right) \sum _{k=1}^{n}(y_k-\bar{{{\mathcal {Y}}}})\right] _{ i \in \{1,\ldots ,p_2\}}\\ \left[ \sigma ^{'}(0)^{l-2} \left( -\textbf{w}_l\ldots W_{3;:i}\right) \sum _{k=1}^{n}(y_k-\bar{{{\mathcal {Y}}}})\right] _{i \in \{1,\ldots ,p_3\}}\\ \vdots \\ \left[ \sigma ^{'}(0)^{2} \left( -\textbf{w}_l\ldots W_{l-1;:i}\right) \sum _{k=1}^{n}(y_k-\bar{{{\mathcal {Y}}}})\right] _{ i \in \{1,\ldots ,p_{l-1}\}}\\ \left[ \sigma ^{'}(0) \left( -\textbf{w}_{l;i}\right) \sum _{k=1}^{n}(y_k-\bar{{{\mathcal {Y}}}})\right] _{ i \in \{1,\ldots ,p_{l}\}}\\ \end{pmatrix}. \end{aligned}$$
  Since $\sum _{k=1}^{n}(y_k-\bar{{{\mathcal {Y}}}})=0$, many entries of the gradient are zero. Let ${\mathscr {D}}_{l}$ is the set $\left\{ (W_2,\ldots ,W_{l-1},\textbf{w}_l) :\ \Vert W_{h;i:}\Vert _2 \right. \left. =1\ \textrm{for}\ i \in \{1,\ldots ,p_{h+1}\}, h\in \{2,\ldots ,l-1\}, \Vert \textbf{w}_l\Vert _2\right. \left. =1\right\}$, where $W_{h;i:}$ is the i-th row of $W_h$. We have
  $$\begin{aligned} \begin{aligned}&\sup _{(W_2,\ldots ,W_{l-1},\textbf{w}_l) \in {\mathscr {D}}_{l}} \left\| \nabla _{\varvec{\theta }_1}{{{\mathcal {L}}}_n({{\mathcal {Y}}}, \mu _{\varvec{\theta }^{0}}({{\mathcal {X}}}))}\right\| _{\infty }\\&=\sup _{(W_2,\ldots ,W_{l-1},\textbf{w}_l) \in {\mathscr {D}}_{l}}\\&\max _{i,j}\left\{ \frac{\sigma ^{'}(0)^{l-1}}{\Vert {{\mathcal {Y}}}_\bullet \Vert _2} \left| \textbf{w}_lW_{l-1}\ldots W_{2;:i}\right| \cdot \right. \\&\left. \left| \sum _{k=1}^{n}(y_k-\bar{{{\mathcal {Y}}}})x_{k,j}\right| \right\} \\&=\frac{\sigma ^{'}(0)^{l-1} \Vert {{\mathcal {X}}}^{\textrm{T}} {{\mathcal {Y}}}_\bullet \Vert _\infty }{\Vert {{\mathcal {Y}}}_\bullet \Vert _2} \sup _{(W_2,\ldots ,W_{l-1},\textbf{w}_l) \in {\mathscr {D}}_{l}} \\&\max _{i}\left\{ \left| \textbf{w}_lW_{l-1}\ldots W_{2;:i}\right| \right\} . \end{aligned} \end{aligned}$$
  Now, we concentrate on $\textrm{supmax}_l=\sup _{(W_2,\ldots ,W_{l-1},\textbf{w}_l) \in {\mathscr {D}}_{l}} \max _{i}\left\{ \left| \textbf{w}_lW_{l-1}\ldots W_{2;:i}\right| \right\}$. When $l=2$, then ${\mathscr {D}}_{2}=\{\textbf{w}_2 | \Vert \textbf{w}_2\Vert _2=1 \}$, so $\textrm{supmax}_l=\sup _{\textbf{w}_2 \in {\mathscr {D}}_{2}}\max _{i}|w_{2,i}|=1=\tau _2$.
  When $l=3$, ${\mathscr {D}}_{3}=\{ (W_2,\textbf{w}_3) | \Vert W_{2;i:}\Vert _2=1, i \in \{1,\ldots ,p_3\}, \Vert \textbf{w}_3\Vert _2=1\}.$ Then, $\textrm{supmax}_l=\sup _{(W_2,\textbf{w}_3) \in {\mathscr {D}}_{3}}\max _{i} |\textbf{w}_3W_{2;:i}|$. Because the $W_{2;:i}$ is the i-th column of $W_2$ and the $l_2$ norm of every row is 1, the maximum is reached by setting one column of $W_2$ to $\textbf{1}_{p_3 \times 1}$ and setting its all other elements to zero. Then $\textrm{supmax}_l=\max _{\Vert \textbf{w}_{3}\Vert _2=1}\sum _{i=1}^{p_3}w_{3,i}=\sqrt{p_3}=\tau _3 \quad \text{( }Cauchy-Schwarz inequality).$ Likewise, the result generalizes to *l*-layers, and we get that
  $$\begin{aligned}{} & {} \sup _{(W_2,\ldots ,W_{l-1},\textbf{w}_l) \in {\mathscr {D}}_{l}} \max _{i}\left\{ \left| \textbf{w}_lW_{l-1}\ldots W_{2;:i}\right| \right\} \\{} & {} =\sqrt{p_3p_4\ldots p_l}=\tau _l. \end{aligned}$$
  the closed form expression for $\lambda _0({{\mathcal {Y}}}, {{\mathcal {X}}})$ of Theorem 3 for regression.
- Classification with cross-entropy loss
  $$\begin{aligned} \begin{aligned}&{{\mathcal {L}}}_n({{\mathcal {Y}}}, \mu _{\varvec{\theta }}({{\mathcal {X}}}))\\&\quad =-\sum _{k=1}^{n}{\varvec{y}}_{k}^{\textrm{T}}\log {\varvec{\pi }}_{k}\\&\quad =-\sum _{k=1}^{n}\sum _{t=1}^{m}y_{k,t}\left[ c_t+W_{l;t:}\sigma (\textbf{b}_{l-1}\right. \\&\quad \left. +W_{l-1}\sigma (\cdots \sigma (\textbf{b}_{1}+W_1{\varvec{x}}_k)))\right. \\&\quad -\log \big \{\sum _{h=1}^{m}\exp \big \{c_h+W_{l;h:}\sigma (\textbf{b}_{l-1}\\&\quad \left. +W_{l-1}\sigma (\cdots \sigma (\textbf{b}_{1}+W_1{\varvec{x}}_k)))\big \}\big \}\right] , \end{aligned} \end{aligned}$$
  where $W_{l;h:}$ is the h-th row in $W_{l}$. Under condition $L_0$, the MLE is $(\hat{\varvec{\theta }_1}, \hat{\textbf{c}})=(\textbf{0}, \log {\bar{{\mathcal {Y}}})}$ for classification, the derivatives with respect to every element are:
  $$\begin{aligned}{} & {} \frac{\partial {{\mathcal {L}}}_n({{\mathcal {Y}}}, \mu _{\varvec{\theta }}(\mathcal{X}))}{\partial W_{1;i,j}}\bigg |_{L_0}\\{} & {} \quad = \sigma ^{'}(0)^{l-1}\sum _{k=1}^{n}x_{k,j}\left( \bar{\mathcal{Y}}-{\varvec{y}}_k\right) ^{\textrm{T}}W_l \ldots W_{2;:i},\\{} & {} \frac{\partial {{\mathcal {L}}}_n({{\mathcal {Y}}}, \mu _{\varvec{\theta }}({\varvec{x}}))}{\partial b_{h;i}}\bigg |_{L_0}\\{} & {} \quad = \sigma ^{'}(0)^{l-h}\sum _{k=1}^{n}\left( \bar{\mathcal{Y}}-{\varvec{y}}_k\right) ^{\textrm{T}}W_l\ldots W_{h+1;:i}, \text { } 1 \\{} & {} \quad \le h \le l-2,\\ {}{} & {} \frac{\partial {{\mathcal {L}}}_n({{\mathcal {Y}}}, \mu _{\varvec{\theta }}({\varvec{x}}))}{\partial b_{l-1;i}}\bigg |_{L_0}\\ {}{} & {} \quad = \sigma ^{'}(0)\sum _{k=1}^{n}\left( \bar{{{\mathcal {Y}}}}-{\varvec{y}}_k\right) ^{\textrm{T}}W_{l;:i}, \end{aligned}$$
  leading to
  $$\begin{aligned}{} & {} \nabla _{\varvec{\theta }_1} {{\mathcal {L}}}_n({{\mathcal {Y}}}, \mu _{\varvec{\theta }^{0}}({\varvec{x}}))\\{} & {} \quad = \begin{pmatrix} \left[ \sigma ^{'}(0)^{l-1}\sum _{k=1}^{n}x_{k,j}\left( \bar{\mathcal{Y}}-{\varvec{y}}_k\right) ^{\textrm{T}}W_l \ldots W_{2;:1}\right] _{ j \in \{1,\ldots ,p_1\}}\\ \vdots \\ \left[ \sigma ^{'}(0)^{l-1}\sum _{k=1}^{n}x_{k,j}\left( \bar{\mathcal{Y}}-{\varvec{y}}_k\right) ^{\textrm{T}}W_l \ldots W_{2;:p_2}\right] _{ j \in \{1,\ldots ,p_1\}} \\ \left[ \sigma ^{'}(0)^{l-1}\sum _{k=1}^{n}\left( \bar{\mathcal{Y}}-{\varvec{y}}_k\right) ^{\textrm{T}}W_l\ldots W_{2;:i}\right] _{ i \in \{1,\ldots ,p_2\}}\\ \left[ \sigma ^{'}(0)^{l-2}\sum _{k=1}^{n}\left( \bar{\mathcal{Y}}-{\varvec{y}}_k\right) ^{\textrm{T}}W_l\ldots W_{3;:i}\right] _{ i \in \{1,\ldots ,p_3\}}\\ \vdots \\ \left[ \sigma ^{'}(0)^{2}\sum _{k=1}^{n}\left( \bar{\mathcal{Y}}-{\varvec{y}}_k\right) ^{\textrm{T}}W_lW_{l-1;:i}\right] _{ i \in \{1,\ldots ,p_{l-1}\}}\\ \left[ \sigma ^{'}(0)\sum _{k=1}^{n}\left( \bar{\mathcal{Y}}-{\varvec{y}}_k\right) ^{\textrm{T}}W_{l;:i}\right] _{ i \in \{1,\ldots ,p_l\}}\\ \end{pmatrix}. \end{aligned}$$
  Since $\sum _{k=1}^{n}(\bar{{{\mathcal {Y}}}}-\textbf{y}_k)=\textbf{0}$, many entries of the gradient are zero. Let ${\mathscr {F}}_{l}$ be the set $\left\{ (W_2,\ldots ,W_{l}) :\ \Vert W_{h;i:}\Vert _2=1 \ \textrm{for} \right. \left. \ i \in \{1,\ldots ,p_{h+1}\}, h\in \{2,\ldots ,l\}\right\}$, where $W_{h;i:}$ is the i-th row of $W_h$. Similarly, via choosing $W_{2;:i}=\textbf{1}_{p_3 \times 1}^{\textrm{T}}$, $W_{h_j;i:}^\textrm{T}=1/\sqrt{p_h} \textbf{1}$ for $h=3,\ldots ,l-1; i=1,2,\ldots ,p_{h+1}$, and $W_{l_j;i:}^\textrm{T}=1/\sqrt{p_l}{{\,\textrm{sgn}\,}}\left( \left( \sum _{k=1}^{n}x_{k,j}\left( \bar{{{\mathcal {Y}}}}-{\varvec{y}}_k\right) \right) _i\right) \textbf{1}$ for any $i \in \{1,2,\ldots ,m\}$, we have
  $$\begin{aligned}{} & {} \sup _{(W_2,\ldots ,W_l) \in {\mathscr {F}}_{l} } \left\| \nabla _{\varvec{\theta }_1} {{\mathcal {L}}}_n({{\mathcal {Y}}}, \mu _{\varvec{\theta }^{0}}({\varvec{x}}))\right\| _{\infty }\\{} & {} \quad =\sup _{(W_2,\ldots ,W_l) \in {\mathscr {F}}_{l} } \\{} & {} \quad \max _{i,j}\left\{ \sigma ^{'}(0)^{l-1}\left| \sum _{k=1}^{n}x_{k,j} \left( \bar{{{\mathcal {Y}}}}-{\varvec{y}}_k\right) ^{\textrm{T}}W_l \ldots W_{2;:i}\right| \right\} \\{} & {} \quad =\sqrt{p_3\ldots p_l}\sigma ^{'}(0)^{l-1}\\{} & {} \quad \max _{j} \sum _{t=1}^{m} \left| \left( \sum _{k=1}^{n}x_{k,j}\left( \bar{\mathcal{Y}}-{\varvec{y}}_k\right) \right) _t\right| \\{} & {} \quad =\tau _l \sigma '(0)^{l-1} \Vert X^{\textrm{T}} {{\mathcal {Y}}}_\bullet \Vert _\infty . \end{aligned}$$

## C Hessian matrix

The notation is that $W_{k;:i}$ be the *i*-th column of $W_{k}$ and $W_{k;h:}$ is the h-th row of $W_{k}$. We consider a 3-layer network for two tasks:

- Regression with the square-root $\ell _2$-loss
  $$\begin{aligned}{} & {} {{\mathcal {L}}}_n({{\mathcal {Y}}}, \mu _{\varvec{\theta }}({{\mathcal {X}}}))\\{} & {} \quad =\sqrt{\sum _{k=1}^{n}\left( y_k-(c+\textbf{w}_{3}\sigma (\textbf{b}_{2}+W_{2}\sigma (\textbf{b}_{1}+W_1{\varvec{x}}_k))) \right) ^{2}} \\{} & {} \quad =: \sqrt{\sum _{k=1}^{n}G_{k}^{2}}. \end{aligned}$$
  Straightforward calculations lead to
  $$\begin{aligned} \begin{aligned}&\frac{\partial {{\mathcal {L}}}_n({{\mathcal {Y}}}, \mu _{\varvec{\theta }}(\mathcal{X}))}{\partial b_{1,i}}\\&\quad = \frac{1}{ {{\mathcal {L}}}_n({{\mathcal {Y}}}, \mu _{\varvec{\theta }}(\mathcal{X}))}\sum _{k=1}^{n}G_{k}\cdot \frac{\partial G_{k}}{\partial b_{1,i}},\\&\frac{\partial ^{2} {{\mathcal {L}}}_{n}({{\mathcal {Y}}}, \mu _{\varvec{\theta }}({{\mathcal {X}}}))}{\partial b_{1,i}^{2}}\\&\quad = \frac{1}{-{{\mathcal {L}}}_{n}^{2}({{\mathcal {Y}}}, \mu _{\varvec{\theta }}({{\mathcal {X}}}))}\cdot \frac{\partial {{\mathcal {L}}}_n({{\mathcal {Y}}}, \mu _{\varvec{\theta }}({{\mathcal {X}}}))}{\partial b_{1,i}} \sum _{k=1}^{n}G_{k}\cdot \frac{\partial G_{k}}{\partial b_{1,i}}\\&\quad +\frac{1}{ {{\mathcal {L}}}_n({{\mathcal {Y}}}, \mu _{\varvec{\theta }}(\mathcal{X}))}\cdot \sum _{k=1}^{n}\left[ \left( \frac{\partial G_{k}}{\partial b_{1,i}}\right) ^{2}+G_{k}\cdot \frac{\partial ^{2} G_{k}}{\partial b_{1,i}^{2}}\right] , \end{aligned} \end{aligned}$$
  and, for $$i\ne j$$,
  $$\begin{aligned} \begin{aligned} \frac{\partial ^{2} {{\mathcal {L}}}_{n}({{\mathcal {Y}}}, \mu _{\varvec{\theta }}({{\mathcal {X}}}))}{\partial b_{1,i}\partial b_{1,j}}&= \frac{1}{-{{\mathcal {L}}}_{n}^{2}({{\mathcal {Y}}}, \mu _{\varvec{\theta }}({{\mathcal {X}}}))}\cdot \frac{\partial {{\mathcal {L}}}_n({{\mathcal {Y}}}, \mu _{\varvec{\theta }}({{\mathcal {X}}}))}{\partial b_{1,j}} \\&\sum _{k=1}^{n}G_{k}\cdot \frac{\partial G_{k}}{\partial b_{1,i}}+\frac{1}{ {{\mathcal {L}}}_n({{\mathcal {Y}}}, \mu _{\varvec{\theta }}(\mathcal{X}))}\cdot \\&\sum _{k=1}^{n}\left[ \frac{\partial G_{k}}{\partial b_{1,i}}\cdot \frac{\partial G_{k}}{\partial b_{1,j}}+G_{k}\cdot \frac{\partial ^{2} G_{k}}{\partial b_{1,i}\partial b_{1,j}}\right] . \end{aligned} \end{aligned}$$
  Under condition $L_0$ (that is $({\varvec{\theta }_1}, c)=(\textbf{0}, \bar{{\mathcal {Y}}})$), the Hessian with respect to $\textbf{b}_1$ is positive semidefinite since
  $$\begin{aligned} \begin{aligned} \frac{\partial ^{2} {{\mathcal {L}}}_{n}({{\mathcal {Y}}}, \mu _{\varvec{\theta }}({{\mathcal {X}}}))}{\partial \textbf{b}_{1}^2}\bigg |_{L_0}&=\frac{n (\sigma ^{'}(0))^{4}W_{2}^{\textrm{T}}\textbf{w}_3^{\textrm{T}}{} \textbf{w}_3W_{2}}{\Vert {{\mathcal {Y}}}_\bullet \Vert _2}. \end{aligned} \end{aligned}$$
  Similarly, the Hessian with respect to $\textbf{b}_2$ at condition $L_0$ is positive semidefinite since
  $$\begin{aligned} \begin{aligned} \frac{\partial ^{2} {{\mathcal {L}}}_{n}({{\mathcal {Y}}}, \mu _{\varvec{\theta }}({{\mathcal {X}}}))}{\partial \textbf{b}_{2}^2}\bigg |_{L_0}&=\frac{n (\sigma ^{'}(0))^{2}{} \textbf{w}_3^\textrm{T}{} \textbf{w}_3}{\Vert {{\mathcal {Y}}}_\bullet \Vert _2}. \end{aligned} \end{aligned}$$
- Classification with cross-entropy loss
  $$\begin{aligned} \begin{aligned} {{\mathcal {L}}}_n({{\mathcal {Y}}}, \mu _{\varvec{\theta }}({{\mathcal {X}}}))&=-\sum _{k=1}^{n}{\varvec{y}}_{k}^{\textrm{T}}\log {\varvec{\pi }}_{k}\\&=-\sum _{k=1}^{n}\sum _{t=1}^{m}y_{k,t}\left[ c_t+W_{3;t:}\sigma (\textbf{b}_{2} \right. \\&\quad \left. +W_{2}\sigma (\textbf{b}_{1}+W_1{\varvec{x}}_k))\right. \\&\quad -\log \big \{\sum _{h=1}^{m}\exp \big \{c_h+W_{3;h:}\sigma (\textbf{b}_{2}\\&\left. \quad +W_{2}\sigma (\textbf{b}_{1}+W_1{\varvec{x}}_k))\big \}\big \}\right] . \end{aligned} \end{aligned}$$
  Straightforward calculations lead to
  $$\begin{aligned} \begin{aligned}&\frac{\partial {{\mathcal {L}}}_n({{\mathcal {Y}}}, \mu _{\varvec{\theta }}(\mathcal{X}))}{\partial b_{1,i}}\\&=-\sum _{k=1}^{n}\sum _{t=1}^{m}y_{k,t} \left[ \sigma ^{'}(b_{1,i}+W_{1;i:}{\varvec{x}}_k)W_{3;t:} \right. \\&\left. \left( \sigma ^{'}(\textbf{b}_2 +W_{2}\sigma (\textbf{b}_{1}+W_1{\varvec{x}}_k))\otimes W_{2;:i}\right) \right. \\&\left. -\frac{\Pi _1}{\sum _{h=1}^{m}\exp \left\{ c_h+W_{3;h:}\sigma (\textbf{b}_{2}+W_{2}\sigma (\textbf{b}_{1}+W_1{\varvec{x}}_k))\right\} } \right] , \end{aligned} \end{aligned}$$
  where $\otimes$ is the Hadamard product and
  $$\begin{aligned} \begin{aligned} \Pi _1=&\sum _{h=1}^{m}\exp \left\{ c_h+W_{3;h:}\sigma (\textbf{b}_{2}+W_{2}\sigma (\textbf{b}_{1}+W_1{\varvec{x}}_k))\right\} \cdot \\&\sigma ^{'}(b_{1,i}+W_{1;i:}{\varvec{x}}_k)W_{3;h:}\left( \sigma ^{'}(\textbf{b}_2+W_{2}\sigma (\textbf{b}_{1} \right. \\&\left. +W_1{\varvec{x}}_k))\otimes W_{2;:i} \right) . \end{aligned} \end{aligned}$$
  Similarly,
  $$\begin{aligned} \begin{aligned}&\frac{\partial {{\mathcal {L}}}_n({{\mathcal {Y}}}, \mu _{\varvec{\theta }}(\mathcal{X}))}{\partial b_{2,i}} = \\&-\sum _{k=1}^{n}\sum _{t=1}^{m}y_{k,t} \left[ W_{3;ti}\sigma ^{'}( b_{2,i}+W_{2;i:}\sigma (\textbf{b}_{1}+W_1{\varvec{x}}_k))\right. \\&\left. -\frac{\Pi _2}{\sum _{h=1}^{m}\exp \left\{ c_h+W_{3;h:}\sigma (\textbf{b}_{2}+W_{2}\sigma (\textbf{b}_{1}+W_1{\varvec{x}}_k))\right\} } \right] , \end{aligned} \end{aligned}$$
  where
  $$\begin{aligned} \begin{aligned} \Pi _2=&\sum _{h=1}^{m}\exp \left\{ c_h+W_{3;h:}\sigma (\textbf{b}_{2}+W_{2}\sigma (\textbf{b}_{1}+W_1{\varvec{x}}_k))\right\} \cdot \\&W_{3;hi}\sigma ^{'}(b_{2,i}+W_{2;i:}\sigma (\textbf{b}_{1}+W_1{\varvec{x}}_k)) \end{aligned} \end{aligned}$$
  and $W_{3;hi}$ is the element in the h-th row and i-th column of $W_3$.
  Under condition $L_0$ (that is $({\varvec{\theta }_1}, \textbf{c})=(\textbf{0}, \log \{\bar{{\mathcal {Y}}}\}$), the Hessian with respect to $\textbf{b}_1$ is
  $$\begin{aligned} \begin{aligned}&\left[ \frac{\partial ^{2} {{\mathcal {L}}}_{n}({{\mathcal {Y}}}, \mu _{\varvec{\theta }}({{\mathcal {X}}}))}{\partial {b}_{1,i}\partial {b}_{1,j}}\bigg |_{L_0}\right] \\&\quad = \big [n\sigma ^{'}(0)^{4}\big \{\bar{{\mathcal {Y}}}^{\textrm{T}}\{(W_3W_{2;:i})\otimes (W_3W_{2;:j})\} \\&\quad -(\bar{\mathcal{Y}}^{\textrm{T}}W_3W_{2;:i})(\bar{{\mathcal {Y}}}^{\textrm{T}}W_3W_{2;:j})\big \} \big ]; \end{aligned} \end{aligned}$$
  the Hessian with respect to $\textbf{b}_2$ is
  $$\begin{aligned} \begin{aligned}&\left[ \frac{\partial ^{2} {{\mathcal {L}}}_{n}({{\mathcal {Y}}}, \mu _{\varvec{\theta }}({{\mathcal {X}}}))}{\partial {b}_{2,i}\partial {b}_{2,j}}\bigg |_{L_0}\right] \\&\quad = \Big [n\sigma ^{'}(0)^{2}\big \{\sum _{h=1}^{m} \bar{\mathcal{Y}}_{h}W_{3;hi}W_{3;hj} \\&\quad -(\sum _{h=1}^{m} \bar{\mathcal{Y}}_{h}W_{3;hi})(\sum _{h=1}^{m} \bar{{\mathcal {Y}}}_{h}W_{3;hj}) \big \} \Big ], \end{aligned} \end{aligned}$$
  where $\bar{{\mathcal {Y}}}_{h}$ is the h-th element in $\bar{{\mathcal {Y}}}$, for $h \in \{1,\ldots ,m\}$.
  To prove the Hessian matrix $\left[ \frac{\partial ^{2} \mathcal{L}_{n}({{\mathcal {Y}}}, \mu _{\varvec{\theta }}({{\mathcal {X}}}))}{\partial {b}_{1,i}\partial {b}_{1,j}}\bigg |_{L_0}\right]$ is positive semidefinite, let $\varvec{\omega }_i$ be $W_3W_{2;:i}$ and $\varvec{\omega }_j$ be $W_3W_{2;:j}$, and we have
  $$\begin{aligned} \begin{aligned}&\left[ \frac{\partial ^{2} {{\mathcal {L}}}_{n}({{\mathcal {Y}}}, \mu _{\varvec{\theta }}({{\mathcal {X}}}))}{\partial {b}_{1,i}\partial {b}_{1,j}}\bigg |_{L_0}\right] \\&= \left[ n\sigma ^{'}(0)^{4}\left\{ \bar{{\mathcal {Y}}}^\textrm{T}\{\varvec{\omega }_i\otimes \varvec{\omega }_j\}-(\bar{\mathcal{Y}}^{\textrm{T}}\varvec{\omega }_i)(\bar{{\mathcal {Y}}}^\textrm{T}\varvec{\omega }_j)\right\} \right] \\&=n\sigma ^{'}(0)^{4} \left[ \varvec{\omega }_i^{\textrm{T}} \left\{ {{\,\textrm{diag}\,}}\{\bar{{\mathcal {Y}}}\}-\bar{{\mathcal {Y}}}\bar{{\mathcal {Y}}}^{\textrm{T}} \right\} \varvec{\omega }_j \right] \\&=n\sigma ^{'}(0)^{4}W_2^{\textrm{T}} W_3^{\textrm{T}}\left[ {{\,\textrm{diag}\,}}\{\bar{\mathcal{Y}}\}-\bar{{\mathcal {Y}}}\bar{{\mathcal {Y}}}^{\textrm{T}}\right] W_3W_2. \end{aligned} \end{aligned}$$
  This Hessian is positive semi-definite provided $\left[ {{\,\textrm{diag}\,}}\{\bar{{\mathcal {Y}}}\} \right. \left. -\bar{{\mathcal {Y}}}\bar{{\mathcal {Y}}}^{\textrm{T}}\right]$ is positive semi-definite. To show this, for any vector $\textbf{x} \in {\mathbb {R}}^{m}$, we have
  $$\begin{aligned} \begin{aligned} \textbf{x}^{\textrm{T}}\left[ {{\,\textrm{diag}\,}}\{\bar{{\mathcal {Y}}}\}-\bar{{\mathcal {Y}}}\bar{{\mathcal {Y}}}^{\textrm{T}}\right] \textbf{x}&=\textbf{x}^{\textrm{T}}{{\,\textrm{diag}\,}}\{\bar{{\mathcal {Y}}}\}{} \textbf{x}-\textbf{x}^{\textrm{T}}\bar{{\mathcal {Y}}}\bar{{\mathcal {Y}}}^{\textrm{T}}{} \textbf{x}\\&=\sum _{i=1}^{m}x_i^{2}\bar{\mathcal{Y}}_{i} -\left( \sum _{i=1}^{m}x_i^{2}\bar{{\mathcal {Y}}}_{i}^{2}\right) ^2 \\&\quad \ge 0 \quad \text {(Cauchy-Schwarz inequality)}. \end{aligned} \end{aligned}$$
  Similarly, the Hessian matrix with respect to $\textbf{b}_2$ is positive semi-definite.

## D Proof of Property 2

Let ${{\mathcal {U}}}\subset {{\mathbb {R}}}$ be any subset of ${{\mathbb {R}}}$. For all $u \in {{\mathcal {U}}}$, choosing $b=\max _{u \in {{\mathcal {U}}}} -u=-\min ({{\mathcal {U}}})$, we have $u=\sigma _{\textrm{ReLU}} (u+b)-b$. Consider the data matrix *X*, $\textbf{u}=X{\varvec{\beta }} \in {\mathbb R}^n$ and $b=-\min _{i=1,\ldots ,n} u_i<\infty$. Then for any point $\textbf{x}\in {{\mathbb {R}}}^{p_1}$ in the convex hull of the *n* data vectors $\{\textbf{x}_i\}_{i=1,\ldots ,n}$, we have $b+\textbf{x}^\textrm{T}{\varvec{\beta }}=\sum _{i=1}^n \lambda _i (b+\textbf{x}_i^\textrm{T}{\varvec{\beta }}) >0$, so a linear function $\mu _{\varvec{\theta }}^{\textrm{lin}}(\textbf{x})=\beta _0+ \textbf{x}^\textrm{T}{\varvec{\beta }} = \beta _0 +\sigma _{\textrm{ReLU}} (\textbf{x}^\textrm{T}{\varvec{\beta }}+b)-b$ can be written as an ANN with a single neuron.

## References

[CR1] Adcock, B., Brugiapaglia, S., Dexter, N., Morage, S.: Deep neural networks are effective at learning high-dimensional Hilbert-valued functions from limited data. In: Proceedings of the 2nd Mathematical and Scientific Machine Learning Conference, vol. 145, pp. 1–36. PMLR (2022)

[CR2] Adcock B, Dexter N (2021). The gap between theory and practice in function approximation with deep neural networks. SIAM J. Math. Data Sci..

[CR3] Advani MS, Saxe AM, Sompolinsky H (2020). High-dimensional dynamics of generalization error in neural networks. Neural Netw..

[CR4] Arlot S, Celisse A (2010). A survey of cross-validation procedures for model selection. Stat. Surv..

[CR5] Bach F, Jenatton R, Mairal J, Obozinski G (2011). Optimization with sparsity-inducing penalties. Found. Trends Mach. Learn..

[CR6] Barron AR (1993). Universal approximation bounds for superpositions of a sigmoidal function. IEEE Trans. Inf. Theory.

[CR7] Bastounis, A., Hansen, A.C., Vlacic, V.: The mathematics of adversarial attacks in AI—why deep learning is unstable despite the existence of stable neural networks. arXiv:2109.06098 (2021b)

[CR8] Bastounis, A., Hansen, A.C., Vlavcic, V.: The extended Smale’s 9th problem—on computational barriers and paradoxes in estimation, regularisation, computer-assisted proofs and learning (2021a)

[CR9] Beck A, Teboulle M (2009). A fast iterative shrinkage-thresholding algorithm for linear inverse problems. SIAM J. Imag. Sci..

[CR10] Belloni A, Chernozhukov V, Wang L (2011). Square-root lasso: pivotal recovery of sparse signals via conic programming. Biometrika.

[CR11] Bölcskei H, Grohs P, Kutyniok G, Petersen P (2019). Optimal approximation with sparsely connected deep neural networks. SIAM J. Math. Data Sci..

[CR12] Breiman L (2001). Random forests. Mach. Learn..

[CR13] Breiman L, Friedman J, Olshen R, Stone C (1984). Classification and Regression Trees.

[CR14] Bühlmann P, van de Geer S (2011). Statistics for High-Dimensional Data: Methods, Theory and Applications.

[CR15] Bühlmann P, Kalisch M, Meier L (2014). High-dimensional statistics with a view toward applications in biology. Ann. Rev. Stat. Appl..

[CR16] Candès EJ, Tao T (2005). Decoding by linear programming. IEEE Trans. Inf. Theory.

[CR17] Carreira-Perpinan, M.A., Idelbayev, Y.: Learning-compression algorithms for neural net pruning. In: 2018 IEEE/CVF Conference on Computer Vision and Pattern Recognition, pp. 8532–8541 (2018)

[CR18] Chao, S.K., Wang, Z., Xing, Y., Cheng, G.: Directional pruning of deep neural networks. In: NeurIPS (2020)

[CR19] Chen T, Chen H (1995). Universal approximation to nonlinear operators by neural networks with arbitrary activation functions and its application to dynamical systems. IEEE Trans. Neural Netw..

[CR20] Chen SS, Donoho DL, Saunders MA (1999). Atomic decomposition by basis pursuit. SIAM J. Sci. Comput..

[CR21] Colbrook MJ, Antun V, Hansen AC (2022). The difficulty of computing stable and accurate neural networks: on the barriers of deep learning and Smale’s 18th problem. Proc. Natl. Acad. Sci..

[CR22] Collins, M.D., Kohli, P.: Memory bounded deep convolutional networks. arXiv:1412.1442 (2014)

[CR23] Curci, S., Mocanu, D.C., Pechenizkiyi, M.: Truly sparse neural networks at scale. arXiv:2102.01732 (2021)

[CR24] Cybenko G (1989). Approximation by superpositions of a sigmoidal function. Math. Control Signals Syst. (MCSS).

[CR25] Descloux P, Sardy S (2021). Model selection with lasso-zero: adding straw in the haystack to better find needles. J. Comput. Graph. Stat..

[CR26] Donoho DL (2006). Compressed sensing. IEEE Trans. Inf. Theory.

[CR27] Donoho DL, Johnstone IM (1994). Ideal spatial adaptation by wavelet shrinkage. Biometrika.

[CR28] Donoho DL, Tanner J (2010). Precise undersampling theorems. Proc. IEEE.

[CR29] Donoho DL, Johnstone IM, Kerkyacharian G, Picard D (1995). Wavelet shrinkage: asymptopia?. J. R. Stat. Soc. B.

[CR30] Donoho DL, Maleki A, Montanari A (2011). The noise-sensitivity phase transition in compressed sensing. IEEE Trans. Inf. Theory.

[CR31] Evci, U., Pedregosa, F., Gomez, A.N., Elsen, E.: The difficulty of training sparse neural networks. arXiv:1906.10732 (2019)

[CR32] Feng, J., Simon, N.: Sparse-input neural networks for high-dimensional nonparametric regression and classification. arXiv:1711.07592 (2019)

[CR33] Friedman JH, Stuetzle W (1981). Projection pursuit regression. J. Am. Stat. Assoc..

[CR34] Friedman JH, Hastie T, Tibshirani R (2010). Regularization paths for generalized linear models via coordinate descent. J. Stat. Softw..

[CR35] Geiger, M., Jacot, A., Spigler, S., Gabriel, F., Sagun, L., d’Ascoli, S., Biroli, G., Hongler, C., Wyart, M.: Scaling description of generalization with number of parameters in deep learning. arXiv:1901.01608 (2019)

[CR36] Giacobino C, Sardy S, Diaz Rodriguez J, Hengardner N (2017). Quantile universal threshold. Electron. J. Stat..

[CR37] Grohs, P., Perekrestenko, D., Elbrächter, D., Bölcskei, H.: Deep neural network approximation theory. arXiv:1901.02220 (2019)

[CR38] Hastie, T., Montanari, A., Rosset, S., Tibshirani, R.J.: Surprises in high-dimensional ridgeless least squares interpolation. arXiv:1903.08560 (2019) 10.1214/21-aos2133 PMC9481183 36120512

[CR39] He J, Jia X, Xu J, Zhang L, Zhao L (2020). Make $$\ell _1$$ regularization effective in training sparse CNN. Comput. Optim. Appl..

[CR40] Hinton, G., Srivastava, N., Krizhevsky, A., Sutskever, I., Salakhutdinov, R.: Improving neural networks by preventing co-adaptation of feature detectors. arXiv:1207.0580 (2012)

[CR41] Hoerl AE, Kennard RW (1970). Ridge regression: biased estimation for nonorthogonal problems. Technometrics.

[CR42] Holland PW (1973). Covariance stabilizing transformations. Ann. Stat..

[CR43] Johnstone IM, Silverman B (2004). Needles and straw in haystacks: empirical Bayes estimates of possibly sparse sequences. Ann. Stat..

[CR44] Kostadinov, D., Voloshynovskiy, S., Ferdowsi, S.: Learning overcomplete and sparsifying transform with approximate and exact closed form solutions. In: 2018 7th European Workshop on Visual Information Processing (EUVIP), pp. 1–6 (2018)

[CR45] Lee, H., Battle, A., Raina, R., Ng, A.Y.: Efficient sparse coding algorithms. In: Proceedings of the 19th International Conference on Neural Information Processing Systems, NIPS’06, pages 801–808. MIT Press (2006)

[CR46] Li Y, Chen CY, Wasserman WW (2016). Deep feature selection: theory and application to identify enhancers and promoters. J. Comput. Biol..

[CR47] Ma, R., Miao, J., Niu, L., Zhang, P.: Transformed $$\ell _1$$ regularization for learning sparse deep neural networks. arXiv:1901.01021 (2019) 10.1016/j.neunet.2019.08.015 31499353

[CR48] Mei S, Montanari A (2021). The generalization error of random features regression: precise asymptotics and double descent curve. Commun. Pure Appl. Math..

[CR49] Mollaysa, A., Strasser, P., Kalousis, A.: Regularising non-linear models using feature side-information. In: Proceedings of the 34th International Conference on Machine Learning, volume 70 of Proceedings of Machine Learning Research, pp. 2508–2517, Sydney (2017)

[CR50] Poggio T, Mhaskar H, Rosasco L, Miranda B, Liao Q (2017). Why and when can deep-but not shallow-networks avoid the curse of dimensionality: a review. Int. J. Autom. Comput..

[CR51] Ranzato, M.A., Boureau, Y.L., LeCun, Y.: Sparse feature learning for deep belief networks. In: Proceedings of the 20th International Conference on Neural Information Processing Systems, NIPS’07, pp. 1185–1192. Curran Associates Inc. (2007)

[CR52] Ravishankar, S., Wen, B., Bresler, Y.: Online sparsifying transform learning-part I: algorithms. IEEE J. Sel. Top. Signal Process. **9**(4), 625–636 (2015) 10.1109/TIP.2018.2865684 30130189

[CR53] Rumelhart DE, Hinton GE, Williams RJ (1986). Learning representations by back-propagating errors. Nature.

[CR54] Srivastava N, Hinton G, Krizhevsky A, Sutskever I, Salakhutdinov R (2014). Dropout: a simple way to prevent neural networks from overfitting. J. Mach. Learn. Res..

[CR55] Sun Y, Song Q, Liang F (2021). Consistent sparse deep learning: theory and computation. J. Am. Stat. Assoc..

[CR56] Tibshirani R (1996). Regression shrinkage and selection via the lasso. J. R. Stat. Soc. B.

[CR57] Ye, M., Sun, Y.: Variable selection via penalized neural network: a drop-out-one loss approach. In: Proceedings of the 35th International Conference on Machine Learning, vol. 80, pp. 5620–5629 (2018)

[CR58] Yuan M, Lin Y (2006). Model selection and estimation in regression with grouped variables. J. R. Stat. Soc. B.

